# Explainable AI improves task performance in human–AI collaboration

**DOI:** 10.1038/s41598-024-82501-9

**Published:** 2024-12-28

**Authors:** Julian Senoner, Simon Schallmoser, Bernhard Kratzwald, Stefan Feuerriegel, Torbjørn Netland

**Affiliations:** 1 ETH Zurich, Zurich, Switzerland; 2 LMU Munich, Munich, Germany; 3 Munich Center for Machine Learning (MCML), Munich, Germany; 4 Present Address: EthonAI, Zurich, Switzerland

**Keywords:** Explainable AI, Task performance, Decision-making, Human-centered AI, Human–AI collaboration, Mechanical engineering, Skin manifestations, Computer science

## Abstract

Artificial intelligence (AI) provides considerable opportunities to assist human work. However, one crucial challenge of human–AI collaboration is that many AI algorithms operate in a black-box manner where the way how the AI makes predictions remains opaque. This makes it difficult for humans to validate a prediction made by AI against their own domain knowledge. For this reason, we hypothesize that augmenting humans with explainable AI improves task performance in human–AI collaboration. To test this hypothesis, we implement explainable AI in the form of visual heatmaps in inspection tasks conducted by domain experts. Visual heatmaps have the advantage that they are easy to understand and help to localize relevant parts of an image. We then compare participants that were either supported by (a) black-box AI or (b) explainable AI, where the latter supports them to follow AI predictions when the AI is accurate or overrule the AI when the AI predictions are wrong. We conducted two preregistered experiments with representative, real-world visual inspection tasks from manufacturing and medicine. The first experiment was conducted with factory workers from an electronics factory, who performed $N=9,600$ assessments of whether electronic products have defects. The second experiment was conducted with radiologists, who performed $N=5,650$ assessments of chest X-ray images to identify lung lesions. The results of our experiments with domain experts performing real-world tasks show that task performance improves when participants are supported by explainable AI with heatmaps instead of black-box AI. We find that explainable AI as a decision aid improved the task performance by 7.7 percentage points (95% confidence interval [CI]: 3.3% to 12.0%, $P=0.001$) in the manufacturing experiment and by 4.7 percentage points (95% CI: 1.1% to 8.3%, $P=0.010$) in the medical experiment compared to black-box AI. These gains represent a significant improvement in task performance.

## Introduction

Artificial intelligence (AI) provides considerable opportunities to assist human work in various domains^1,2^. For example, in manufacturing, AI is widely used to support humans when inspecting the quality of produced products to identify defects^3^. Similarly, in medicine, disease diagnosis now makes increasing use of AI systems. For instance, a recent survey found that AI is used by about 15% of radiologists at least weekly^4^. More broadly, an analysis showed that about 30% of all jobs in the United States are at high exposure to be assisted by AI^5^. Hence, the importance of human–AI collaboration is expected to grow in the near future.

However, many questions regarding the effective design of human–AI collaborations remain open. One particular challenge in the use of AI for human work is that state-of-the-art AI algorithms, which frequently involve millions of trainable parameters^6,7^, operate as “black-box” algorithms. The term “black-box” refers to the opacity of these systems, meaning that the internal workings and decision-making processes of these algorithms are not transparent or easily understandable by humans^8^. This can have crucial implications in practice as the lack of transparency makes it difficult—or even impossible—for humans to validate the predictions made by an AI against their domain knowledge. Hence, without being able to assess whether a prediction generated by an AI is accurate, humans will not be able to correct predictions of the AI, because of which the unique expertise of workers is essentially lost, which will make the collaboration between domain experts and AI largely ineffective.

Increasing efforts have been made to overcome the black-box nature of AI by developing methods that generate *explanations* for how AI algorithms reach their decisions^9–15^. Explainable AI refers to a set of methods that support humans in understanding how AI algorithms map certain inputs (e.g., lung X-rays, patient characteristics) to certain outputs (e.g., probability estimates for pneumonia)^16,17^. Explainable AI can be broadly categorized into inherently interpretable models and post-hoc explanation techniques (see Supplement A for an extended literature review on explainable AI). For inherently interpretable algorithms, the decision-making of the algorithm can be inspected by humans, e.g., by inspecting the coefficients in linear regression or the splitting rules in decision trees^18^. Post-hoc explanation techniques are required when the inner workings of an AI algorithm become too complex to be understood by humans such as in deep neural networks. For example, one approach is to approximate the behavior of a black-box AI with a simpler model (e.g., a linear model) that can be interpreted^19^. Other methods rely on game theory to estimate the contribution of each model input to the model output while considering possible interaction effects^20^. Such explanation techniques are commonly used by AI engineers in the development of AI algorithms. Therefore, this literature stream is orthogonal to the use of explainable AI in our work, where we use post-hoc explanation techniques to improve decisions by domain experts in real-world job tasks.

Common methods for explaining AI algorithms in computer vision include the use of heatmaps. These heatmaps visually highlight the areas that are most relevant to the predictions made by the AI^21,22^. In this research, we focus on heatmaps because they can easily be understood by humans and already have broad adoption in practice. This choice is motivated by our research setting, which involves visual inspection tasks in manufacturing and medicine where spatially localized explanations are highly relevant. Heatmaps are very common in explaining black-box AI models for visual inspection tasks and are considered state-of-the-art with respect to their localization performance across various settings^23–26^. Another advantage of heatmaps is their ease of use, which is especially relevant in real-world settings where not all domain experts may be familiar with explainable AI methods. These domain experts can then compare the easily interpretable heatmap explanations to their domain knowledge, thereby validating whether the AI is correct or overwriting the AI if it is not correct.

Several works have studied behavioral dimensions of human–AI collaboration. For example, it has been examined whether humans are willing to delegate work to AI^27–29^. Another common dimension is algorithm aversion, where humans are averse to following decisions by algorithms and instead rely on their own (mis)judgment^30–34^. An antecedent to algorithm aversion is *trust in AI*, critically influencing whether humans adopt or reject AI recommendations^35–37^. Oppositely to algorithm aversion, overreliance is also a problem negatively impacting the effectiveness of human–AI collaboration^38–40^. That is, humans risk following AI predictions blindly without attentively performing the task. While all these dimensions are interesting from a behavioral perspective, the main outcome of interest for business and healthcare organizations is *task performance*. However, the impact of explainable AI on task performance in human–AI collaboration in real-world job tasks remains unclear.

We hypothesize that augmenting domain experts with explainable AI through visual heatmaps, as opposed to black-box AI, improves task performance in human–AI collaboration. Specifically, we treat explainable AI as a form of decision aid that supports domain experts in better understanding algorithmic decisions. Here, explainable AI does not provide more information from an AI perspective (i.e., identical predictive performance). However, for domain experts, accessing explainable AI through heatmaps gives rich additional information by making the AI predictions more intelligible. Thus, we expect that domain experts supported by explainable AI through heatmaps will outperform those supported by black-box AI in two ways: (1) they are more likely to follow AI predictions when they are accurate, and (2) they are more likely to overrule AI predictions when they were wrong.

Previous research has studied the effect of explainable AI on task performance in human–AI collaboration (see Supplement A for a detailed overview), yet with key limitations. In particular, existing works are typically restricted by either (i) recruiting laypeople or (ii) overly simplified tasks that are not representative of real job tasks^41–44^. However, a realistic estimate of the effect of explainable AI on task performance requires a real-world task performed by domain experts. Such works that actually study real-world tasks with domain experts are on the other hand restricted by (i) comparing explainable AI vs humans alone^45,46^, (ii) using no real explainable AI^47^, or (iii) research designs that do not isolate the effect of explainable AI on task performance^48–50^. In contrast, the strength of our work is that we study the effect of explainable AI through visual heatmaps on task performance relative to black-box AI in human–AI collaborations with *real-world tasks* and actual *domain experts*.

The objective of this study is to analyze the effect of explainable AI in the form of visual heatmaps (as opposed to black-box AI) on task performance of domain experts performing real-world job tasks. For this, we conducted two preregistered experiments in which domain experts were asked to solve real-world visual inspection tasks in manufacturing (Study 1) and medicine (Study 2). We followed a between-subject design where we randomly assigned participants to two treatments: (a) black-box AI (i.e., where AI predictions are opaque) and (b) explainable AI (i.e., where AI predictions are explained with heatmaps). For simplicity, we later refer to our heatmap explanations as “explainable AI”. The latter thus offers not only the prediction from the AI but also shows explanations in the form of a visual heatmap as a decision aid. Study 1 was conducted in a manufacturing setting, where participants had to identify quality defects in electronic products. For this, we specifically recruited factory workers performing $N=9,600$ assessments of electronic products in a factory setting at *Siemens*. Study 2 was conducted in a medical setting, where participants had to identify lung lesions on chest X-ray images. To that end, medical professionals, i.e., radiologists, were recruited and performed $N=5,650$ assessments of chest X-ray images. In both studies, participants performed better when being supported by explainable AI as a decision aid.

The tasks of both experiments are representative of many real-world human–AI collaborations. The manufacturing task is an identical, one-to-one copy of a real-world job task at *Siemens* and, hence, highly representative of visual inspection tasks in manufacturing^51,52^. Visual inspection tasks are standard in the manufacturing industry. Regardless of how much manufacturers have sought to build quality into products and processes, labor-intensive inspection tasks still abound^53^. In healthcare, visual inspection tasks are common across many different subdisciplines, such as dermatology, radiology, pathology, ophthalmology, and dentistry, among many others. As concrete examples, physicians have to inspect, for instance, skin lesions in dermatology, tissues in pathology, and lung lesions in radiology^26,54,55^. Therefore, establishing whether physicians benefit from explainable AI in visual inspection tasks is highly relevant for disease diagnosis.

## Methods

We conducted two randomized experiments across two different visual inspection settings: manufacturing (Study 1) and medicine (Study 2). In both experiments, participants had to perform a visual inspection task. We preregistered our hypotheses (i.e., Study 1: https://osf.io/7djxb and Study 2: https://osf.io/69yqt; see also Supplement K), which were tested in two randomized experiments. The research design was approved by the Ethics Commission of ETH Zurich (EK 2021-N-34). We confirm that all methods were performed in accordance with the relevant guidelines and regulations. All participants provided informed consent.

### Experiment design

In the following, details of the experiment designs of the two visual inspection tasks are provided.

#### Study 1: manufacturing experiment

For the manufacturing task, we designed a representative, real-world visual inspection task in collaboration with *Siemens Smart Infrastructure* in Zug, Switzerland. The experimental task is representative of various domains in which workers have to make decisions under a limited time budget. During the experiment, workers were shown images of electronic products and were asked to label them as faultless or defective. Reassuringly, we emphasize that our experiment involved a real work scenario: we conducted it with real workers familiar with quality management practice, a real user interface for state-of-the-art quality management, realistic incentives, and real product images. All steps in the experiments were carried out via a computer interface that was designed analogously to the real-world quality inspection setup at *Siemens* (see Supplement D for details). In the experiment, we made sure that all workers have the exact same conditions (exact same product images, same computer setup, same time limits, etc.). Thereby, we can rule out confounding variables that would arise naturally during the usual work routines and thus ensure that the experiment is scientifically sound.

We obtained 200 images of four different types of electronic products (printed circuit boards) from *Siemens*. Example images are provided in Supplement B. All four different product types are of equal importance to *Siemens*. Each product type comprised 43 images with faultless products and 7 images with defective products (e.g., missing components, wrong components, and faulty components). The different defects are all considered equally bad by the partner company, i.e., the products are considered to be either functional or non-functional. Hence, we considered defective products as scrap as best practice in quality management^51,52^.

We implemented an AI algorithm that computed an individual quality score for each image. The quality score gives a numerical value between 0 (most certainly defect) and 100 (most certainly faultless). Workers were instructed that a quality score below 90 suggests an increased likelihood of a quality defect and that the AI algorithm can make mistakes. As humans cannot understand how the AI algorithm arrives at the prediction, the quality score is regarded as opaque (“black-box AI”). When evaluating the quality score with a cutoff of 90 for mapping the numerical value onto a binary faultless/defect label, the standalone AI algorithm achieves a balanced accuracy of 95.6% and a defect detection rate of 92.9%. The prediction performance of the standalone AI algorithm was not communicated to the workers. The AI algorithm was trained on an additional set of product images that was not included in the experiment.

We used anomaly heatmaps^23^ to explain the opaque quality score from the AI algorithm. The heatmaps were computed with standard computer vision methods and highlighted image regions with suspected quality defects (i.e., deviations from a faultless product). We chose heatmaps as the explanation technique for our AI algorithm as they provide a clear and intuitive way of highlighting areas with quality defects. This is especially important since the recruited domain experts are typically not familiar with explanation techniques for AI algorithms. Further, heatmaps are frequently used for images and are considered state-of-the-art with respect to their localization performance across various settings^23–26^. Details on the implementation of the AI algorithm and heatmaps are provided in Supplement C.

**Figure 1:**
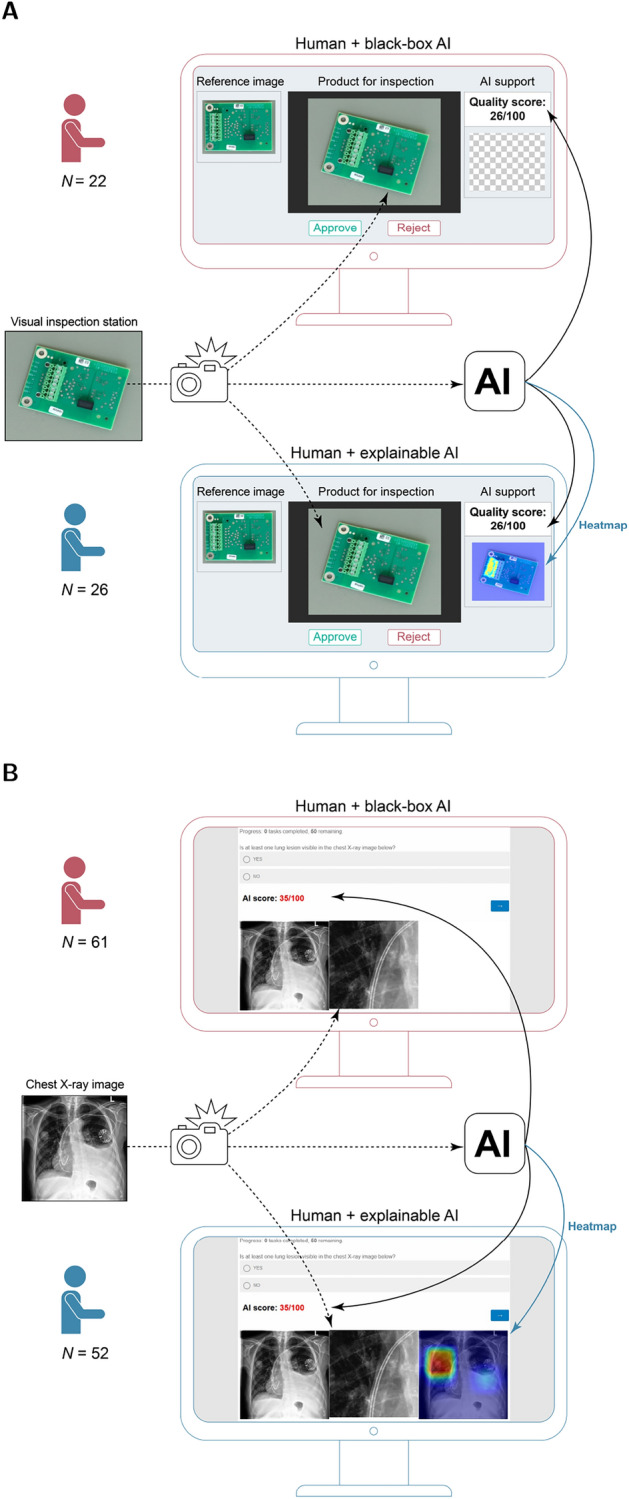
Overview of the experiments for assessing the effect of explainable AI on task performance. ( A ) Experimental design of the manufacturing experiment where factory workers were asked to “approve” images of faultless products and to “reject” images of defective products through a computer interface. ( B ) Experimental design of the medical experiment where radiologists were asked to decide whether lung lesions are visible in the chest X-ray image. In both experiments, participants were randomly assigned to one of the two treatments: (**a**) black-box AI or (**b**) explainable AI.

In the experiment, workers were randomly assigned to one of the two treatments: (a) black-box AI or (b) explainable AI in the form of visual heatmaps (Figure 1A). Workers in the black-box AI treatment arm were only supported by the opaque quality score. Workers in the explainable AI treatment arm had access to the same quality score but additionally received the heatmap that explained the otherwise opaque quality score. Of note, the explainable AI had the same accuracy as the black-box AI and did not carry more information from an AI perspective (i.e., the heatmaps were of the same predictive power).

Before starting the experiment, workers had to give written consent to participate and then pass a tutorial on how to use the interface. After that, the workers were randomly assigned to one of the two treatments, i.e., either black-box AI or explainable AI. During the experiment, the 200 product images were consecutively shown in random order. For each image, the workers had to assess the quality; that is, to “approve” or “reject” the shown product. We tracked the decision speed and the quality assessment (i.e., labeled as faultless or defective) made by the worker. To match real-world conditions, the workers were given a maximum of 35 minutes to finish the inspection task of 200 product images (around 10 seconds per image). In total, $N=9,600$ assessments of product images were performed by the workers. Finally, workers completed a post-experimental questionnaire (Supplement L).

#### Study 2: medical experiment

For the medical task, radiologists had to identify lung lesions in real chest X-ray images. Lung lesions are common findings in chest X-ray images^56^ and can be easily overlooked due to their frequently small size^57^. The radiologists were asked whether at least one lung lesion was visible in the X-ray image. The experiment was conducted via Qualtrics. To ensure a realistic experimental setup that resembles the same task in daily medical practice, we implemented a zoom function, which allowed the radiologists to investigate an enlarged view of the image by moving their computer mouse over the image. Analogous to Study 1, we emphasize that our medical experiment involved a realistic work scenario: we conducted it with actual medical professionals, who were asked to investigate real chest X-ray images.

We used 50 chest X-ray images from the CheXpert dataset^58^. The dataset comprised 7 images with at least one lung lesion and 43 images without lung lesions. Example images are provided in Supplement B.

We implemented an AI algorithm that outputs the probability of whether a lung lesion is visible in the chest X-ray image. We transformed these probability outputs for lung lesions such that the AI score gives a numerical value between 0 (most certainly contains a lung lesion) and 100 (most certainly does not contain a lung lesion) to mirror the quality score from the manufacturing setting. As a result, the AI output can be interpreted as a risk score, which is widely used in medical practice. Radiologists were instructed that an AI score below 90 indicates that the AI algorithm suspects at least one lung lesion is visible and that the AI algorithm can make mistakes. When evaluating the AI score with a cutoff of 90 for mapping the numerical value onto a binary label (lung lesion visible yes/no), the standalone AI algorithm achieves a balanced accuracy of 82.2% and a disease detection rate of 71.4%. Analogously to the manufacturing task, the prediction performance of the standalone AI algorithm was not communicated to the participants.

As in the manufacturing task, the black-box AI algorithm was converted into an explainable AI by explaining the AI score via a heatmap, which is a state-of-the-art explanation technique for chest X-ray images in medicine^26^. Further details about the implementation of the AI algorithm and the heatmap are provided in Supplement C.

The procedure was analogous to the manufacturing experiment. Before starting the experiment, radiologists had to confirm their area of specialization and give written consent to participate. Subsequently, the task was explained and the radiologists had to pass a tutorial on how to use the interface. After that, radiologists were randomly assigned to one of the two treatments, i.e., either black-box AI or explainable AI (Figure 1B). During the experiment, 50 chest X-ray images were randomly shown either in forward or reverse order. For each chest X-ray image, the radiologists had to answer whether at least one lung lesion is visible. The corresponding answers as well as the decision speed were tracked. Radiologists were given a maximum of 35 minutes to finish the inspection task of 50 chest X-ray images. Radiologists were given more time per image compared to factory workers in the manufacturing experiment to reflect the differences in manufacturing and clinical practice. In total, $N=5,650$ assessments of chest X-ray images were performed by the radiologists. Finally, all radiologists completed a post-experimental questionnaire (Supplement L).

### Study populations

The inclusion was as follows. Participants had to be at least 18 years old. For the manufacturing task, participants additionally had to have no self-reported visual impairment. The exclusion criteria were preregistered and were as follows. In both studies, we excluded participants who failed the tutorial or did not finish the inspection task on time. Participants with obvious misbehavior were also excluded from our analyses. In the manufacturing task, this was the case for workers who approved all products (i.e., labeled no images as defective). In the medical task, this was the case for radiologists who assigned the same label for all 50 chest X-ray images. In both studies, participants whose performance with respect to balanced accuracy was more than three standard deviations worse than the mean of their respective treatment arm were excluded. We performed randomization checks to confirm that all treatment arms were demographically unbiased (Supplement E).

#### Study 1: manufacturing experiment

The manufacturing experiment was carried out from June 29 to July 8, 2021, on-site at a *Siemens* factory in Zug, Switzerland. The objective of the field experiment was to get a real-world estimate of the treatment effect based on a representative sample of actual factory workers. Therefore, we only considered factory workers who were experienced in quality control practices. The factory workers were well familiar with the shown products and the visual inspection task. Overall, 56 factory workers (consisting of manufacturing employees, quality engineers, and team leaders) participated in our study. Out of them, all workers passed the tutorial; 6 did not finish on time and 2 were excluded due to obvious misbehavior. The final sample consisted of 48 factory workers with an average working experience of 13.8 years ($SD=9.8$). A larger sample size in our manufacturing experiment was not possible because the entire available workforce in one shift did not exceed 56 workers. Still, the experiment is well-powered as the treatment effect in the field experiment is considerably large. No additional financial incentive was given beyond the base salary to be representative of many real-world tasks from domain experts (e.g., as in manufacturing at *Siemens*).

#### Study 2: medical experiment

The medical experiment was carried out via an online interface from February 27 to March 31, 2024. Actual radiologists based in the United States were recruited via MSI-ACI (https://site.msi-aci.com/). MSI-ACI paid a financial compensation to radiologists regardless of performance and adheres to the federal minimum wage in the United States. Overall, 122 radiologists started the study. Out of them, all passed the tutorial; 4 did not complete the study and 2 did not finish on time; and 3 were excluded due to obvious misbehavior. Thus, the final sample consisted of 113 radiologists with an average tenure as radiologist of 13.5 years ($SD=10.4$).

### Statistical analysis

In manufacturing, it is common that all defects (e.g., missing components, wrong components, and faulty components) are considered equally bad^51,52^, so that the product to inspect could be either functional or non-functional. Analogously, in the medical setting, either lung lesions were present or not. Hence, in both settings, the outcomes were binary. Therefore, task performance in the visual inspection tasks between the participants’ assessments and the ground-truth labels was computed via (1) balanced accuracy (i.e., average sensitivity across faultless and defective products) and (2) defect/disease detection rate. For (1), the balanced accuracy is calculated via $0.5 \times [ TP /P + TN /N]$ with true positives $TP$, positives *P*, true negatives $TN$, and negatives *N*. Here, we used balanced accuracy since it accounts for imbalanced distributions of labels by equally weighing the performance on each label, thus following best practice^59^. In contrast, the standard accuracy score would not account for the imbalanced distribution of positive and negative labels encountered in both settings (i.e., 172 faultless products and 28 defective products in the manufacturing setting; 7 chest X-ray images with and 43 without lung lesions in the medical setting). For (2), the defect/disease detection rate is defined as $TN /N$, where defective products and chest X-ray images with lung lesions were defined as negatives. In our manufacturing setting, missing a defective product has more severe implications than labeling a faultless product as defective. In medicine, missing a lung lesion on a chest X-ray image has more severe implications than additionally performing a CT scan for a healthy patient. For that reason, it is crucial to find the negative samples.

All statistical tests in the results are based on one-sided Welch’s *t*-tests. We further used ordinary least square (OLS) regression models to estimate the treatment effect of explainable AI on task performance. The OLS models are estimated via

1 $$\begin{aligned} Y_{i} = \beta _{0} + \beta _{1}\, Treatment _{i} + \varepsilon _{i}, \end{aligned}$$

where $Y_{i}$ is the observed task performance (i.e., balanced accuracy or defect/disease detection rate), $Treatment_{i}$ is a binary variable which equals 0 if participant *i* received the black-box AI treatment and 1 if participant *i* received the explainable AI treatment. The *P*-values for $\beta _{1}$ were derived using Student’s t-test. A significance level of $\alpha = 0.05$ was preregistered.

All analyses were conducted using Python (3.11) with *numpy* (1.24.3)^60^, *pandas* (1.5.3)^61^, *scipy* (1.11.1)^62^, and *statsmodels* (0.14.0)^63^. The data visualizations were created with *seaborn* (0.12.2)^64^ and *matplotlib* (3.7.1)^65^.

### Robustness checks

To assess whether our findings are also generalizable to non-experts, we repeated the manufacturing task with participants recruited from Amazon MTurk (Results and Supplement J).

Additionally, we conducted the following robustness checks. First, we repeated our analyses using precision as an additional task performance metric (Supplement G). Second, we repeated the OLS regression models with additional participant-specific controls to estimate the treatment effect of explainable AI (Supplement H). Third, we estimated the treatment effect with quasi-binomial regression (Supplement H). Fourth, we estimated the regression models including participants that were previously excluded due to obvious misbehavior or because they did not finish the inspection task in time (Supplement I). All robustness checks yielded conclusive findings.

To demonstrate that the heatmaps in our medical setting are robust with respect to the choice of algorithm, we used two additional, different algorithms to generate heatmaps. We find that different algorithms lead to similar heatmaps (Supplement F).

## Results

To analyze the effect of explainable AI on task performance in human–AI collaboration, we conducted two randomized experiments across two different settings, i.e., in manufacturing (Study 1) and medicine (Study 2). In both experiments, participants had to perform a visual inspection task. In the manufacturing experiment, factory workers were asked to inspect electronic products and to identify defective products. In the medical experiment, radiologists were asked to decide whether lung lesions are visible in chest X-ray images. Participants were randomly assigned to one of two different treatments aiding them in the task: (a) black-box AI or (b) explainable AI (Figure 1). Participants with black-box AI received an opaque AI score as a decision aid. Participants with explainable AI received the same score and an additional decision aid: the explanation of the score in the form of a heatmap. The heatmap does not provide more information from an AI perspective (the score is identical) but allows users to verify the prediction made by the AI. However, heatmaps provide a clear and intuitive way of highlighting quality defects/lung lesions^66^. We hypothesized that explainable AI as a decision aid improves task performance of domain experts in human–AI collaboration.

### Study 1: manufacturing experiment

**Figure 2:**
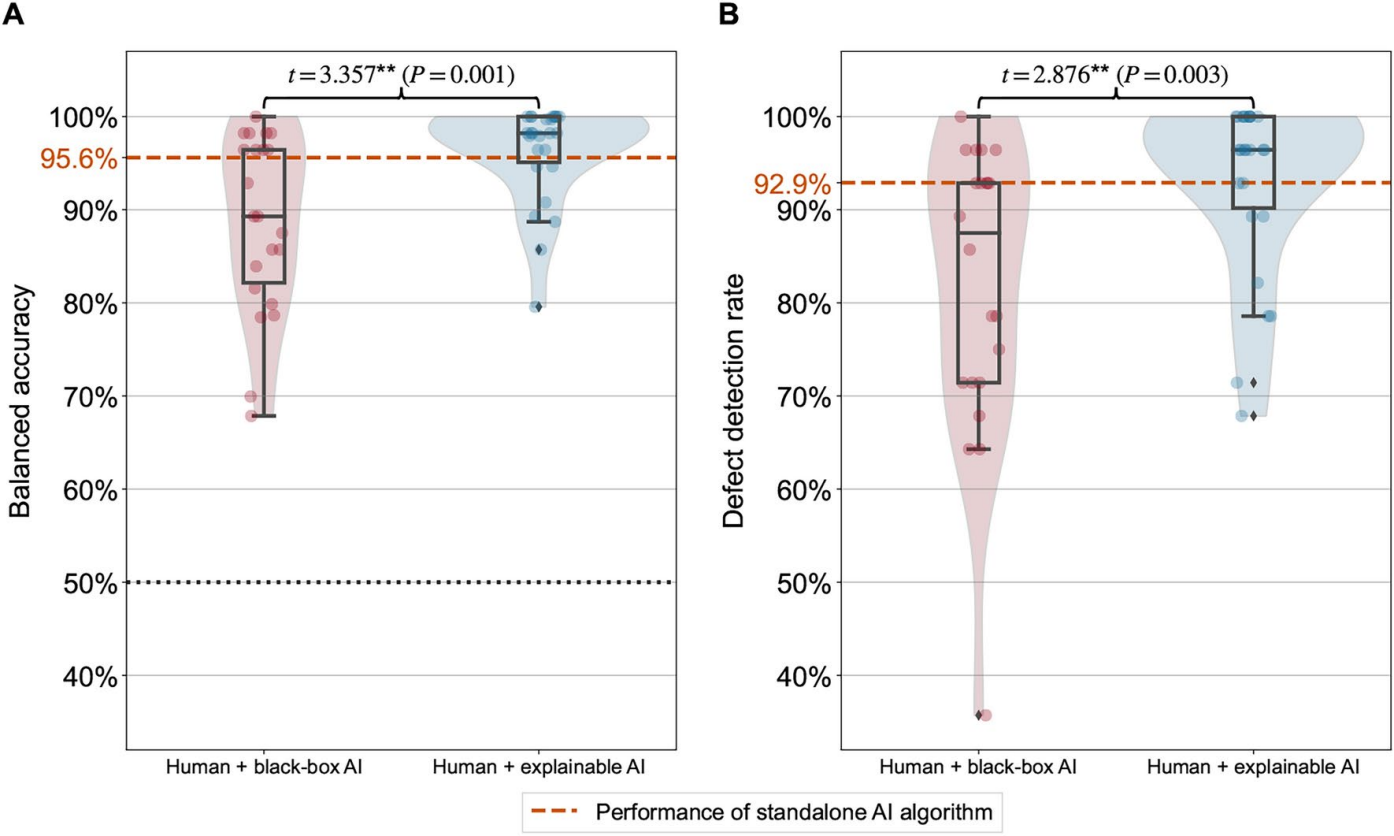
Results of manufacturing experiment. The boxplots compare the task performance between the two treatments: black-box AI and explainable AI. The task performance is measured by the balanced accuracy (**A**) and the defect detection rate (**B**) based on the quality assessment of workers and the ground-truth labels of the product images. A balanced accuracy of 50% provides a naïve baseline corresponding to a random guess (black dotted line). The standalone AI algorithm attains a balanced accuracy of 95.6% and a defect detection rate of 92.9% (orange dashed lines). Statistical significance is based on a one-sided Welch’s *t*-test (^***^$P<0.001$, ^**^$P<0.01$, ^*^$P<0.05$). In the boxplots, the center line denotes the median; box limits are upper and lower quartiles; whiskers are defined as the 1.5x interquartile range.

In the manufacturing experiment, factory workers were asked to identify quality defects in electronic products (e.g., missing components, wrong components, and faulty components) with high accuracy. We found that workers supported by explainable AI achieved better task performance than workers supported by black-box AI. Workers with black-box AI achieved a balanced accuracy with a mean of only 88.6%, whereas workers with explainable AI treatment achieved a balanced accuracy with a mean of 96.3% (Fig. 2A). We then estimated the treatment effect of explainable AI by regressing the balanced accuracy on the treatment (black-box AI $= 0$, explainable AI $= 1$). The regression results show that the treatment effect of explainable AI is statistically significant and large ($\beta =7.653$, $SE =2.178$, $t = 3.513$, $P=0.001$, 95 % $\text {confidence interval [CI]} = [3.268, 12.038]$); that is, an improvement of 7.7 percentage points. Compared to the black-box AI, the explainable AI leads to a five-fold decrease in the median error rate.

Workers with explainable AI outperformed workers with black-box AI also with respect to the defect detection rate with a mean of 93.0% versus a mean of 82.0% (Fig. 2B). The regression results again confirm that the treatment effect of explainable AI is statistically significant and large ($\beta =11.014$, $SE =3.680$, $t = 2.993$, $P=0.004$, 95 % $\text {CI} = [3.607, 18.421]$). All regression results remain statistically significant when including relevant control variables (demographics, tenure, self-reported IT skills, and decision speed) in the regression model (see Supplement H).

A detailed analysis of the workers’ assessments revealed that workers with explainable AI followed accurate predictions more often than workers with black-box AI ($\text {mean} = 93.5\%$ for black-box AI, $\text {mean} = 98.6\%$ for explainable AI). In particular, workers supported by black-box AI were 3.6 times more likely to erroneously overrule an AI prediction, despite the prediction being accurate ($t=2.437$, $P=0.011$). Interestingly, 73.1% of the workers with explainable AI performed even better than the standalone AI algorithm. This suggests that the explanations (i.e., the heatmaps) not only improve adherence to accurate AI predictions, but also help humans make correct assessments when the AI predictions are wrong. We found that workers with explainable AI were, on average, able to identify and overrule 96.9% of the wrong AI predictions. For comparison, workers supported by black-box AI only overruled 86.4% of the wrong AI predictions. These results are highly relevant since – regardless of an AI’s performance—wrong AI predictions can always occur due to external factors such as dust or different light conditions. The difference between both treatments is again statistically significant ($t=2.631$, $P=0.007$). These findings underscore the effectiveness of augmenting humans with explainable AI.

Finally, we assessed whether workers with explainable AI spent more time on making their quality assessments. For this, we analyzed whether the workers’ median decision speeds across the 200 product images differed. No statistically significant difference ($t=0.308$, $P=0.380$) was observed between both treatments ($\text {mean} = {5.01}\,{\textrm{s}}$ for black-box AI, $\text {mean} = {4.88}\,{\textrm{s}}$ for explainable AI). Therefore, explainable AI improved task performance without affecting the productivity of the workers.

### Study 2: medical experiment

In the medical experiment, radiologists were asked to visually inspect 50 chest X-ray images and decide whether at least one lung lesion was visible (Fig. 1B). Visual inspection tasks like ours are common in medicine across various subdisciplines^54,55^.

**Figure 3:**
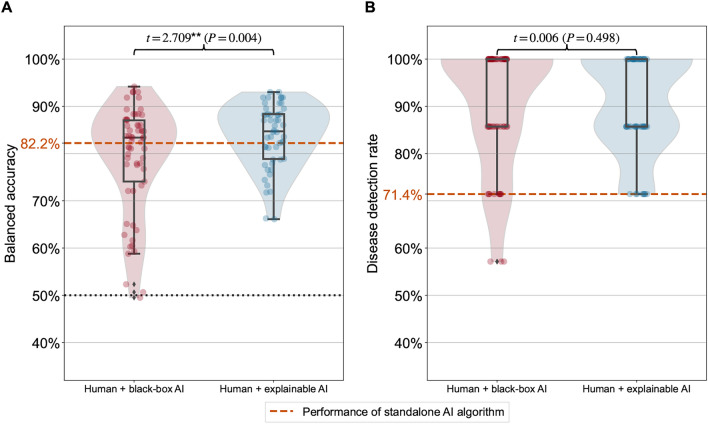
Results of medical experiment. The boxplots compare the task performance between the two treatments: black-box AI and explainable AI. The task performance is measured by the balanced accuracy (**A**) and the disease detection rate (**B**) based on the quality assessment of radiologists and the ground-truth labels of the chest X-ray images. A balanced accuracy of 50% provides a naïve baseline corresponding to a random guess (black dotted line). The standalone AI algorithm attains a balanced accuracy of 82.2% and a disease detection rate of 71.4% (orange dashed lines). Statistical significance is based on a one-sided Welch’s *t*-test (^***^$P<0.001$, ^**^$P<0.01$, ^*^$P<0.05$). In the boxplots, the center line denotes the median; box limits are upper and lower quartiles; whiskers are defined as the 1.5x interquartile range.

Radiologists augmented with explainable AI outperformed peers with black-box AI. Radiologists with black-box AI achieved a balanced accuracy with a mean of only 79.1%, whereas radiologists with explainable AI achieved a balanced accuracy with a mean of 83.8% (Fig. 3A). We again estimated the treatment effect of explainable AI by regressing the balanced accuracy on the treatment (black-box AI $= 0$, explainable AI $= 1$). The regression results show that the treatment effect of explainable AI is statistically significant and large ($\beta =4.693$, $SE =1.800$, $t = 2.608$, $P=0.010$, 95 % $\text {CI} = [1.127, 8.259]$); that is, an improvement of 4.7 percentage points. All results remain statistically significant when including relevant control variables (tenure, self-reported IT skills, and decision speed) in the regression model (see Supplement H). In contrast to the manufacturing experiment, no difference in task performance with respect to the disease detection rate was observed; radiologists in both treatment arms achieved a disease detection rate with a mean of 90.4% (Fig. 3B). This was also observed when regressing the disease detection rate on the treatment ($\beta =-0.014$, $SE =2.244$, $t = -0.006$, $P=0.995$, 95 % $\text {CI} = [-4.460, 4.433]$). This can be expected since missing a lung lesion has more serious consequences than erroneously believing a lung lesion is visible; thus, leading to conservative decision-making of radiologists. Therefore, we additionally inspected precision as a task performance metric. We find that radiologists augmented with explainable AI were significantly more precise (improvement of 6.4 percentage points, $P=0.014$) in identifying lung lesions compared to radiologists with black-box AI (see Supplement G).

As in Study 1, we found that radiologists with explainable AI followed accurate AI predictions more often than radiologists with black-box AI treatment ($\text {mean} = 72.4\%$ for black-box AI, $\text {mean} = 82.1\%$ for explainable AI). In particular, radiologists supported by black-box AI were 54.2% times more likely to erroneously overrule an AI prediction, although it was correct ($t=3.084$, $P=0.001$). We observed that radiologists with explainable AI only overruled 50.8% of the wrong AI predictions compared to 57.7% for radiologists with black-box AI treatment.

Again, we assessed the decision speed of radiologists in both treatment arms. We found no significant difference ($t=0.392$, $P=0.348$) between both treatments ($\text {mean} = {10.71}\,{\textrm{s}}$ for black-box AI, $\text {mean} = {10.29}\,{\textrm{s}}$ for explainable AI). Thus, task performance was improved by explainable AI without reducing the productivity of the radiologists.

### Comparison to non-experts

Additionally, we repeated the manufacturing task with non-experts recruited from Amazon MTurk as a robustness check (Supplement J). Typically, non-experts can not leverage explanations in the same way as domain experts due to missing domain knowledge. Hence, we were interested whether the large treatment effect of explainable AI on task performance we observed with domain experts transfers also to non-experts.

We found that non-experts supported by explainable AI also achieved a higher task performance than non-experts supported by black-box AI. Task performance of non-experts augmented with explainable AI was improved by 6.3 percentage points with respect to balanced accuracy. However, the treatment effect of explainable AI is slightly smaller as compared to the experiment with domain experts (where the balanced accuracy increased by 7.7 percentage points). Furthermore, non-experts with explainable AI achieved a higher defect detection rate with an improvement of 11.3 percentage points. This is of a similar effect size as in the real-world experiment (11.0 percentage points). The treatment effects in both metrics were again statistically significant (balanced accuracy: $\beta =6.252$, $SE =1.733$, $t = 3.608$, $P<0.001$, 95 % $\text {CI} = [2.841, 9.664]$; defect detection rate: $\beta =11.271$, $SE =3.276$, $t = 3.440$, $P=0.001$, 95 % $\text {CI} = [4.822, 17.720]$). For more details, see Supplement J.

## Discussion

The “age of AI” redefines the way humans and machines collaborate, thus raising questions about how human–AI collaborations can be effectively designed. As we show, the effectiveness of human–AI collaboration largely depends on the extent to which humans incorporate correct AI predictions and overrule wrong ones. However, many state-of-the-art AI algorithms operate as black-box, thus making it difficult for humans to compare the reasoning of the AI to their own domain knowledge. In this paper, we contribute a unique perspective by studying the impact of AI explainability through heatmaps on task performance of domain experts in human–AI collaboration, presenting empirical evidence from different domains with robust and generalizable results.

### Interpretation

We conducted two preregistered experiments to estimate the effect of explainable AI through heatmaps in human–AI collaboration in real-world visual inspection tasks. Our results demonstrate that domain experts make subpar decisions when they are supported by a black-box AI algorithm with opaque predictions. In contrast, we find that explanations from an explainable AI are a powerful decision aid. Explanations were provided in the form of visual heatmaps, which provide a clear and intuitive way of highlighting areas that are determinants of AI predictions and, additionally, have been shown to perform well in localizing relevant parts of an image^23–26^. The explanations do not provide more information from an AI perspective (i.e., the prediction performance is identical), but rather make the information more accessible to domain experts. Specifically, compared to black-box AI, augmenting domain experts with visual heatmaps improved the task performance by 7.7 percentage points (95% CI: 3.3% to 12.0%, $P=0.001$) in a manufacturing experiment and by 4.7 percentage points (95% CI: 1.1% to 8.3%, $P=0.010$) in a medical experiment. In the manufacturing experiment, 73.1% of the domain experts even outperformed the standalone AI algorithm when they were augmented with explainable AI through heatmaps. The prime reason was that domain experts supported by explainable AI were more likely to follow AI predictions when they were accurate and more likely to overrule them when they were wrong.

Our work contributes experimental evidence to the literature on human–AI collaboration^30–32,67–75^. Cognitive biases of humans in the use of AI may impede decision-making processes and further provide a barrier to the wider adoption of human–AI collaboration. For example, overreliance and algorithm aversion are two common cognitive biases that refer to the decision-making processes and the adoption of AI systems, respectively. To counteract these, prior literature has presented several remedies, such as describing the functional logic of an algorithm^70^, giving users permission to modify an algorithm^31^, letting users integrate their own forecasts into an algorithm^72^, or cognitive forcing functions that force humans to analytically engage with the AI recommendations^76^. Our paper presents evidence of an effective alternative; that is, explaining individual predictions from an otherwise opaque AI algorithm. Such explanations allow domain experts to validate how an AI arrives at a certain prediction. Naturally, it is possible that cognitive biases may also extend toward explanations of an AI, which has been reported previously in experiments with non-experts^38^. Yet, a strength of our experiment is that we account for cognitive biases in human decision-making (e.g., overreliance) due to the fact that we measure the overall performance of the actual decisions based on the AI system in the field. Yet, we acknowledge that we can only control for biases in the decision-making processes of domain experts and not biases related to whether managers may make decisions against the adoption of AI systems in the first place (e.g., due to algorithm aversion).

A strength of our study is that we gather empirical evidence of improved task performance by explainable AI through heatmaps compared to black-box AI. In particular, by performing experiments in two different settings, we demonstrate that these results are generalizable. Unlike previous works on studying task performance in human–AI collaboration^41–44^, we (i) conducted experiments of two real-world job tasks in manufacturing and medicine and (ii) recruited domain experts for those tasks, i.e., factory workers and radiologists. When experiments use simplified decision tasks (e.g., object recognition) that are not representative of actual human work in the field, real-world validity is reduced. In contrast, our study has high external validity.

Our work is orthogonal to the literature on explainable AI in computer science, where the main goal is to develop and evaluate new methods for explaining black-box AI algorithms. Contrary, we are interested in a behavioral outcome, namely task performance in human–AI collaboration. A previous study on the effect of explainable AI on task performance found an improvement of 1.5 percentage points in accuracy relative to black-box AI^50^. However, their experiment was designed such that participants were not only shown the real explainable AI but also systemically biased explainable AI. This could have decreased the trust of the participants in the AI and, thus, explain the smaller treatment effect in comparison to our experiments. Prior work has also made use of expert annotations as a proxy for explainable AI^47^. However, this prevents any conclusion on whether real explainable AI improves task performance.

Policy initiatives in many countries aim to promote transparency in AI algorithms (e.g., the United States^77^ and the European Union^78^). These efforts are usually motivated from the perspective of ethics, regulation, and safety^9,79–81^. Our research suggests that the benefits of algorithmic transparency are more profound: augmenting domain experts with explainable AI can enable better decisions with benefits for individuals, organizations, and society.

### Implications

Improving the task performance of domain experts has wide-ranging practical implications across fields—not only in manufacturing and medicine as documented in this work. For example, factory workers at *Siemens* augmented with explainable AI through heatmaps were able to identify 13% more defects than peers augmented with black-box AI. Thus, explainable AI can help reduce costs for manufacturing companies by filtering out defective products at the earliest possible stage. It also assists workers in succeeding at their work tasks, potentially contributing to higher work satisfaction. Similarly, in medicine, task performance of physicians is crucial, especially for important tasks such as identifying possibly cancerogenous lung lesions. By showing that the results are consistent across different settings, we demonstrate that our insights are likely to be generalizable to other visual inspection tasks.

### Limitations

One limitation of our research is that we, in both experiments, studied one specific human–AI work setting (a visual inspection task) with one specific form of explainability (a heatmap indicating the location of potential quality defects or lung lesions). However, this experimental task is representative of many real-world human–AI work settings and heatmaps are standard in explaining AI predictions of images. We also show that other heatmap algorithms lead to similar results (see Supplement F). Nevertheless, it is possible that other explainable AI methods may have a different effect on task performance.

We also acknowledge reservations against using explainable AI in general. Explainable AI can be fooled by adversarial attacks^82^ or may itself generate explanations that are unreliable and thus lead to misleading conclusions^8^. Nevertheless, it is likely that performance improvements from explainable AI can be achieved in other settings, where explanations serve as a decision aid. Further, it is important to note that, in both experiments, 14% of the images contained quality defects/lung lesions. Thus, quality defects/lung lesions were more prevalent than what domain experts would typically encounter in their respective job. This discrepancy might have influenced their prior expectations while performing the task. However, as participants in both treatment arms were shown exactly the same images, this factor likely had minimal impact on our findings.

### Future work

In this study, we focused on two distinct settings, where workers perform visual inspection tasks, namely manufacturing and medicine. Future research may seek to generalize our findings to entirely different settings, such as finance (e.g., fraud detection) or human resources (e.g., candidate screening).

Our study relied on heatmaps as the explainable AI method for images. However, a plethora of different methods exist. For example, counterfactual explanations are another common approach to explain a black-box AI model^83^. There, the goal is to identify minimal changes in the image to alter the prediction of the AI model, allowing a counterfactual image to be compared to the original. This comparison can highlight relevant features, such as lung lesions in X-ray images. Future research could investigate whether different explainable AI methods, such as counterfactual explanations, impact the task performance in human–AI collaboration differently.

Further, the tasks in this work, which had to be performed by domain experts, relied only on unstructured data (i.e., images). However, structured data require different explainable AI methods than unstructured data, because of which heatmaps may not be applicable. We invite future work to further analyze the effect of explainable AI on task performance using appropriate methods for structured data such as Shapley additive explanations (SHAP)^20,75^ or local interpretable model-agnostic explanations (LIME)^19^, which try to estimate the impact of each feature on the predicted outcome.

### Conclusion

This study demonstrates that explainable AI in the form of heatmaps significantly enhances task performance in human–AI collaboration compared to black-box AI. By providing visual heatmaps, explainable AI supports domain experts in making more accurate visual inspection decisions, allowing them to validate AI predictions against their own domain knowledge. This approach led to notable performance improvements in both manufacturing and medical inspection tasks. These findings highlight the potential value of explainable AI in increasing the reliability and utility of human–AI collaboration across fields.

## Supplementary Information

Supplementary Information.

## Data Availability

The code to reproduce the results from all studies is publicly available at https://github.com/simonschallmoser/ExplainableAIimprovesTaskPerformance. Data will be made available by the corresponding author upon reasonable request.
