# Supplementary material for: Explainable AI improves task performance in human–AI collaboration
Source: Sci Rep. 2024 Dec 28;14:31150. doi: 10.1038/s41598-024-82501-9 (PMC11681242; doi:10.1038/s41598-024-82501-9)
Supplement: Supplementary file 1 — Supplementary Information. [file 41598_2024_82501_MOESM1_ESM.pdf]

Explainable AI improves task performance in human-AI collaboration

Julian Senoner, Simon Schallmoser, Bernhard Kratzwald,

Stefan Feuerriegel, Torbjørn Netland

Supplements

Contents

A Extended literature review **4**

A.1 Explainable AI in computer science 4

A.2 Explainable AI in behavioral science 7

B Research setting **9**

B.1 Manufacturing setting 9

B.2 Medical setting 12

C Implementation of AI algorithm **14**

C.1 Manufacturing setting 14

C.2 Medical setting 16

D Experimental interface **19**

D.1 Manufacturing setting 19

D.2 Medical setting 20

E Randomization checks **22**

E.1 Study 1: Manufacturing experiment 22

E.2 Study 2: Medical experiment 22

F Robustness of the heatmap **24**

G Results with precision as task performance metric **26**

H Regression models **28**

H.1 Study 1: Manufacturing experiment 28

H.2 Study 2: Medical experiment 29

I Analysis with excluded participants **31**
I.1 Study 1: Manufacturing experiment . . . 31
I.2 Study 2: Medical experiment . . . 32
J Experiment with non-experts **33**
J.1 Results with precision as task performance metric . . . 35
J.2 Randomization checks . . . 36
J.3 Regression models . . . 37
J.4 Analysis with excluded participants . . . 38
K Preregistered hypotheses **40**
L Post-experimental questionnaire **42**

Supplement A Extended literature review

In the following, we provide an extended literature review of explainable artificial intelligence (AI). In particular, we differentiate research on explainable AI in computer science (which is primarily focused on methodological outcomes) from our work (which is focused on behavioral science outcomes). An overview is provided in Table S1.

A.1 Explainable AI in computer science

In the field of computer science, the primary objective concerning explainable AI is to develop and evaluate new methods to achieve better transparency of AI algorithms. For a general overview of explainable AI, see for example [1, 2, 3, 4]. AI algorithms can be broadly divided into two categories: algorithms that are considered to be inherently interpretable and algorithms that are not due to their complexity [5]. The latter are often referred to as black-box algorithms.

An inherently interpretable model is linear regression, where the decision-making can be directly followed by inspecting the coefficients. Extensions of linear regression are generalized linear models (GLMs) and generalized additive models (GAMs) [6, 7]. GLMs were introduced as a unification of various methods that allow for different distributions of the dependent variable (e.g., a binary dependent variable as in logistic regression). GAMs were introduced to also allow for non-linear relationships between an independent and the dependent variable, which can be modeled for example with decision trees or shallow neural networks (see e.g., [8, 9]). While GLMs and GAMs are still considered to be inherently interpretable, they are not as straightforward to interpret as linear regression.

Decision trees are also considered to be inherently interpretable by simply following the decision rules from the root to the leaf nodes. Other inherently interpretable models are naïve Bayes classifier, k-nearest neighbor algorithm, rule-based learning, etc. (see [10] for an introduction).

In contrast, post-hoc explanation techniques can be applied to better understand black-box algorithms such as neural networks. These explanation techniques are applied after the AI algorithm has been trained. Post-hoc explanation methods can be divided into global and local methods [3], where the former aim at explaining the algorithm's overall decision-making process while the latter provide explanations for a single, specific input. An example of global methods are feature importance rankings, which rank the features based on their importance in predict-

ing a model's outcome, usually measured across the entire model rather than for individual predictions. A further example are partial dependence plots, which show the effect of a single feature on the predicted outcome of a model, averaged over a dataset. Prominent examples of local methods are local interpretable model-agnostic explanations (*LIME*) and SHapley Additive exPlanations (*SHAP*) [11, 12]. *LIME* approximates a black-box model locally around the prediction with an interpretable model (like a linear model) to explain individual predictions. *SHAP* leverages a concept from cooperative game theory (Shapley values [13]) to explain the output of a model by computing the contribution of each feature to the prediction while also considering possible interaction effects. Post-hoc explanation methods can be further categorized into model-specific and model-agnostic methods. Model-specific methods are designed for a specific class of AI algorithms or even for a single AI algorithm. Model-specific methods exist, for example, for convolutional neural networks [14] or for kernel-based AI algorithms such as support vector machines [15]. In contrast, model-agnostic methods can be applied to any AI algorithm; notable examples are *LIME* and *SHAP*. Another dimension to differentiate post-hoc explanation methods is for which data type (tabular, text, audio, images, etc.) the method was developed. In this study, we focus on images, and, in the following, we thus present some of the most relevant post-hoc explanation methods for AI algorithms in computer vision. For general overviews of explainable AI in computer vision, we refer to [16, 17].

In addition to fully interpretable and post-hoc explanation techniques, there also exist methods that do not fall in either of these categories. Prominent examples are the following. One approach, for example, involves transforming a black-box AI model into a white-box model [18, 19], i.e., a model that is inherently interpretable. Another approach is called data canyon and allows for a visual interpretation of a black-box AI model [20].

One of the earliest attempts to explain convolutional neural networks (CNN), which are nowadays widely used in computer vision, was made by Zeiler and Fergus [21]. Therein, the authors present the deconvolutional network (*DeconvNet*) as a visualization method that maps feature activations back to the input image. Additionally, a simple technique called occlusion was discussed as a method for explaining the predictions of a CNN. For that, different portions of the input image are systematically occluded with a grey square, and the impact on the output of the network is observed. Significant changes in the output probabilities indicate the regions of the image most important for classification.

In [22], a method was introduced for generating saliency maps by computing the gradient of the output category with respect to the input image. This technique highlights the regions of the image that contribute most to the model's classification decision, offering a straightforward visual explanation of where the network is "looking" to make its predictions.

Layer-wise relevance propagation *(LRP)* backtracks the output decision of the network through the layers to assign relevance scores to individual pixels. This method helps in understanding which parts of the input image were most relevant for the model's decision, emphasizing a layer-by-layer decomposition of the prediction [23].

Integrated gradients attribute the prediction of a neural network to its input features, calculating the gradients of the output prediction with respect to the input image. It integrates these gradients along the path from a baseline (zero input) to the actual input, offering a way to visualize the importance of each pixel [24].

Deep learning important features (*DeepLIFT*) compares the activation of each neuron to its 'reference activation' and assigns contribution scores according to the difference. This method can identify which features of the input contribute to differences in the output from some baseline, offering a more detailed view than simple gradient-based methods [25].

By using the global average pooling layers in CNNs, class activation mapping (*CAM*) generates heatmaps that highlight the discriminative parts of the image used by the network to identify specific classes, facilitating visual explanations of model decisions [26]. Several extensions to this approach exist [14, 27, 28], with *GradCAM* being one of the most often used approach [14]. In contrast to *CAM*, *GradCAM* employs a gradient-based approach to generate heatmaps, and, as a result, no changes to the network architecture are required.

Counterfactual explanations provide insights by showing how a small change in the input image could change the classification result. This method helps in understanding model decisions by answering "what-if" scenarios, offering a direct way to comprehend how the model might react to different inputs [29].

A special case of post-hoc explanation in computer vision are anomaly heatmaps. These were developed for unsupervised computer vision AI algorithms, i.e., algorithms that do not require a labeled dataset but rather aim at finding anomalies automatically [30, 31, 32].

A plethora of post-hoc explanation methods exists and often it is not obvious which method to choose. Thus, different evaluation measures for post-hoc explanation methods have been

proposed [33]. These include fidelity, which measures how accurately the explanations reflect the decisions of the underlying model [11, 34, 35, 36], and robustness, assessing stability under small changes in the input [37]. Another measure is human-interpretability, which examines how understandable the explanations are to humans [38]. But also application-grounded measures have been proposed, where the evaluation metric is how humans perform in a certain task [39, 40].

In another stream of literature in computer science, tools that help AI engineers with designing and training AI algorithms are developed. Especially, deep neural networks are difficult to train and require a certain amount of experience. Therefore, visualization tools have emerged that facilitate this task (e.g., see [41, 42, 43, 44]).

A.2 Explainable AI in behavioral science

In behavioral science, different outcomes of human-AI collaboration have been studied. Human delegation of tasks and decisions to AI algorithms has been intensively studied recently [45, 46, 47, 48]. Specifically, it has been examined whether the use of explainable AI can increase the likelihood of humans delegating decisions to AI algorithms [49].

Algorithm aversion refers to the phenomenon where humans are reluctant to use algorithms [50, 51, 52, 53, 54, 55, 56, 57, 58, 59, 60, 61, 62]. To overcome human aversion towards AI algorithms, it has been hypothesized that providing an explanation of an AI's decision may be beneficial. This has been tested with mixed findings [63, 64]. Missing trust in an AI's decision can lead to algorithm aversion [65]. Therefore, previous studies investigated whether explainable AI can increase trust in AI algorithms [66, 67, 68, 69, 70, 71, 72, 73, 74, 75, 76, 77].

A contrary phenomenon to algorithm aversion is overreliance [78, 79], where humans place too much trust in AI algorithms, potentially overlooking or ignoring their limitations. Previous research has found mixed results on whether explainability of AI leads to decreased overreliance [80, 79, 81].

However, for a good task performance, it is crucial that humans only adhere to correct AI predictions and overrule wrong ones, which is also referred to as appropriate reliance [82]. The effect of explainable AI on task performance has been studied previously. In the majority of studies, however, non-experts (e.g., via Amazon Mechanical Turk or university students) were recruited and those oftentimes performed simplified, non-realistic, or even non-relevant tasks (see, e.g., [83, 84, 85, 86, 87, 88, 89, 90, 91, 71, 92, 93, 94, 95, 96, 97, 80, 78, 98, 81, 99, 100, 101]).

It has been hypothesized that non-experts can not fully harness explanations due to a lack of domain knowledge [81]. Thus, an empirical evaluation of the effect of explainable AI on task performance in real job tasks, requires actual domain experts of those tasks [33].

Previous studies that recruited domain experts to perform real-job tasks have other drawbacks. For example, prior work has compared the effect of explainable AI against humans alone [102, 103]. Others have used expert annotations as a proxy for explainable AI [104] or research designs that prevent isolating the treatment effect of explainable AI on task performance [39, 105, 77]. Finally, also other outcomes have been studied such as trust, confidence, and perceived usefulness [106, 107], while, as our novelty, we add by focusing on task performance with domain experts.

Table S1: Overview of key literature on explainable AI.

Domain	Concept	Research summary	Dependent variable	References (examples)
Computer science	New explanation methods	Derivations of new methods where the focus is on mathematical / algorithmic contributions	n/a	[6, 7, 8, 9, 11, 12, 15, 21, 22, 23, 24, 25, 26, 14, 27, 28, 29, 30, 31]
	Benchmarking methods/ datasets	Proposing new methods or datasets to benchmark the performance of explainable AI	n/a	[34, 35, 36, 37, 38, 39, 40]
	New visualization tools	Visualization tools that facilitate the development of complex machine learning models	n/a	[41, 42, 43, 44]
Behavioral science	Delegation between humans and AI	Humans avoid delegation to algorithms	Delegation frequency	[45, 46, 47, 48, 49]
	Algorithm aversion	Humans reject advice from algorithm	Adherence	[50, 51, 52, 53, 54, 55, 56, 57, 58, 59, 60, 61, 63, 62, 64]
	Trust in AI	Humans do not trust AI algorithms	Trust	[66, 67, 68, 69, 70, 71, 72, 73, 74, 75, 76, 77]
	Overreliance on AI	Humans follow advice from algorithms blindly	Overreliance	[80, 79, 81]
	Task performance in response to explainable AI	Comparison of black-box AI vs explainable AI for task performance using unrealistic tasks, non-experts, or non-causal research designs	Task performance	[83, 84, 85, 86, 87, 88, 89, 90, 91, 71, 92, 93, 94, 95, 96, 97, 80, 78, 98, 81, 99, 100, 102, 103, 104, 39, 105, 77, 101]
		Real-world job tasks with domain experts for estimating treatment effects of explainable AI vs black-box AI	Task performance	**ours**

Supplement B Research setting

B.1 Manufacturing setting

Poor quality generates 10% to 15% of the operating expenses in manufacturing.[1] Identifying defective products before they move downstream in the value chain is essential to maintain a high operational performance. For this purpose, manufacturers conduct visual quality inspections to assess whether products have defects (e.g., assembly errors or surface damages) [30]. In manufacturing operations, many quality inspections are still conducted manually, which is often a tedious, tiring, and error-prone task. AI offers promising opportunities to overcome these drawbacks by supporting factory workers in automatically detecting quality defects before products are sold to customers. Specifically, AI can assist workers in detecting the location and type of error so rework can be conducted more effectively and efficiently. Therefore, AI algorithms enable factory workers to be more productive by focusing on their key value-creating work tasks.

Our research was carried out at *Siemens* Smart Infrastructure in Zug, Switzerland. To test our hypotheses, the company provided us with real-world product images (each with 1920×1080 pixels) from their factory. The images comprise four different types of electronic products, all of which are printed circuit boards. Figure S1 shows example images of the four types of electronic products that were inspected during the experiment. Figure S2 shows three examples of quality defects, which include products with wrong components, products with assembly errors, and products with faulty components.

Overall, we received two datasets. The first dataset comprised 200 images, including 43 correct products and 7 defective products for each of the four product types. All experiments (and thus the empirical results in the main analysis) are based on the first dataset. The second dataset comprised 200 additional faultless images (50 for each of the four product types). These images were used to train the AI algorithm that was used to compute the quality scores in the experiment (see Supplement C).

[1] American Society for Quality. *Cost of Quality (COQ)*. URL: https://asq.org/quality-resources/cost-of-quality, last accessed on November 18, 2024.

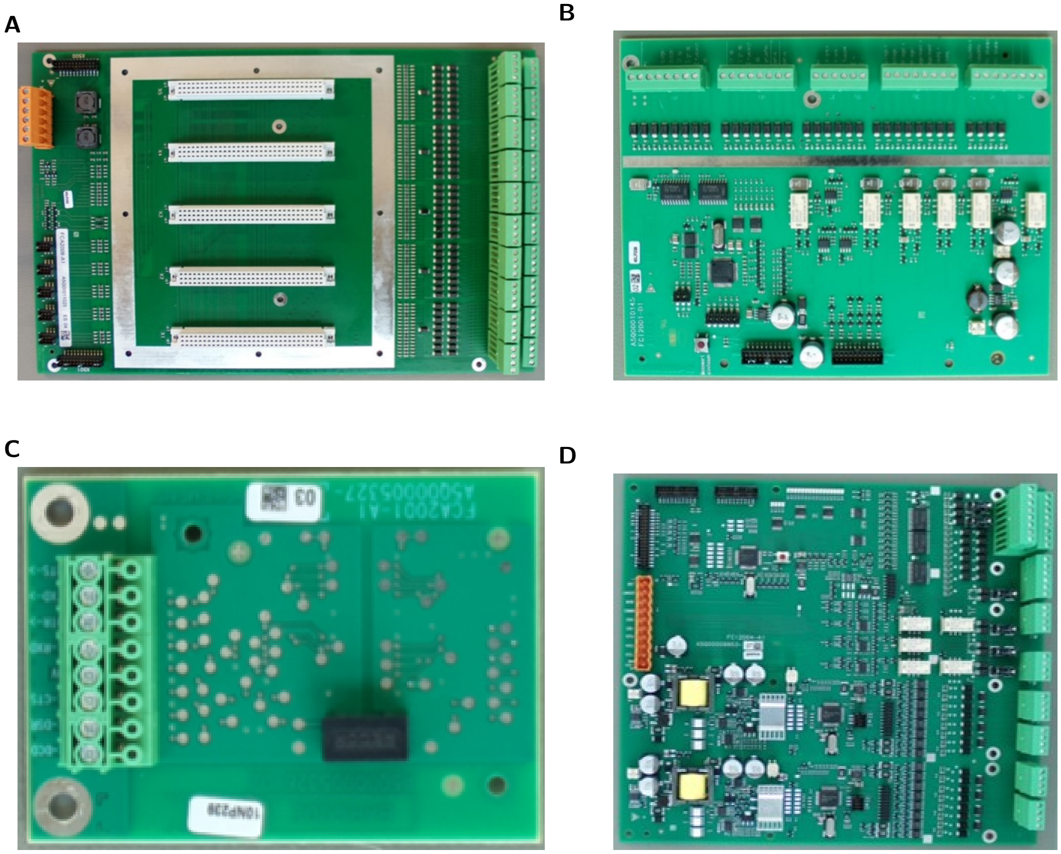

Figure S1: Four types of electronic products (printed circuit boards). (**A**-**D**) Exemplary images of faultless products that were inspected during the experiment.

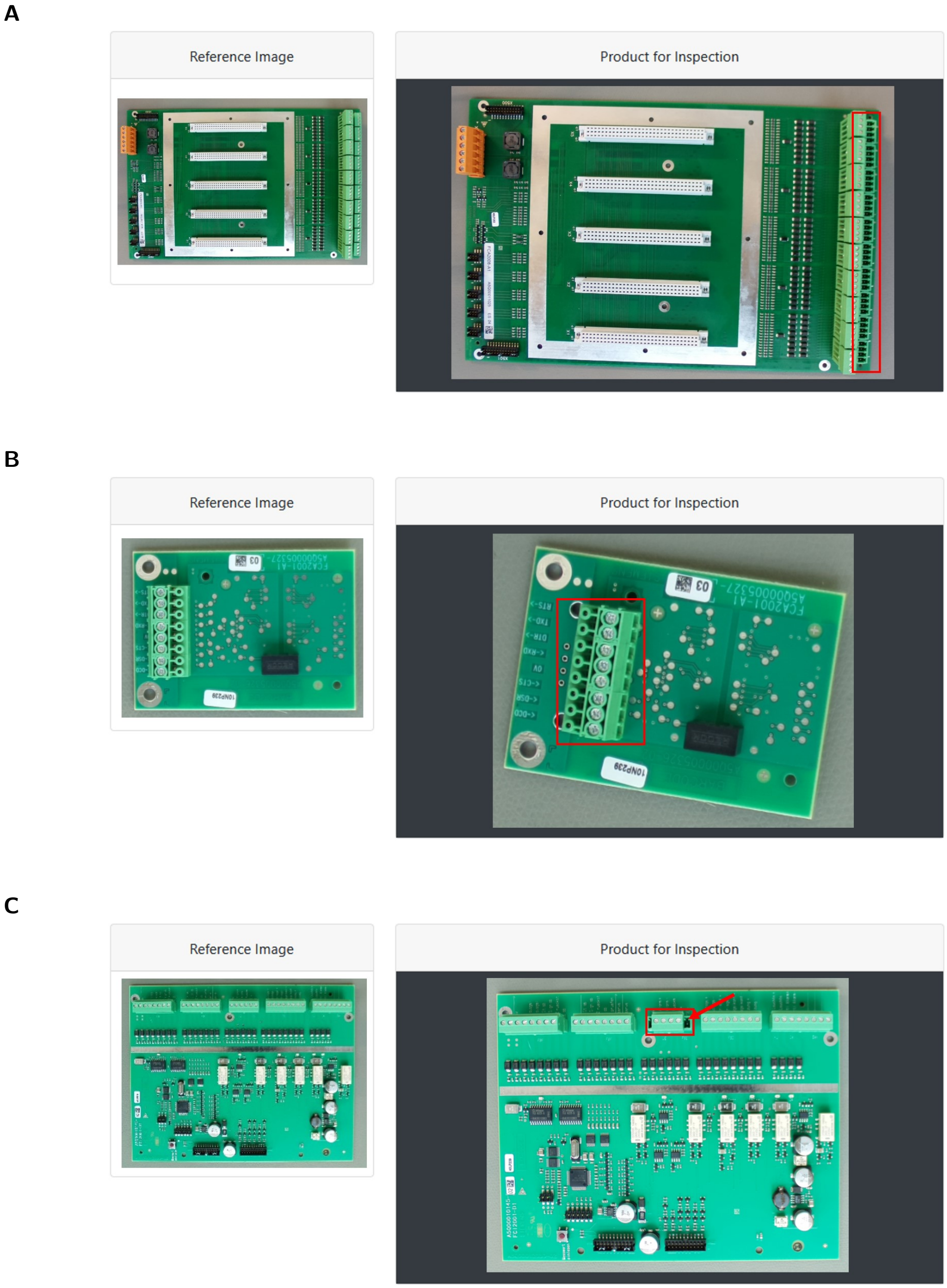

Figure S2: Examples of quality defects. (**A**) Example of a defective product with wrong components. (**B**) Example of a defective product with a component assembled in the wrong orientation. (**C**) Example of a defective product with a faulty component.

B.2 Medical setting

Chest radiography (capturing X-ray images) is a widely performed diagnostic imaging test across the world and plays a crucial role in the screening, diagnosis, and management of numerous diseases that pose a threat to life [108]. One of such diseases are lung lesions, which include lung nodules and masses in our experiment. Lung nodules are common and are encountered on roughly one out of 500 chest X-ray images [109].

Overlooking a lung lesion on a chest X-ray can have serious, potentially life-threatening consequences for patients. The failure to detect a lesion at an early stage can lead to a delay in diagnosis and treatment, allowing diseases to progress to more advanced stages. This can significantly worsen the prognosis for conditions such as lung cancer, tuberculosis, and pneumonia, where early intervention can often lead to better outcomes. Beyond the immediate health risks, there are also implications for patient care, including increased medical costs due to more complex and prolonged treatment that may become necessary as a disease progresses. However, subtle lung lesions can be easily overlooked even without any constraints on how long radiologists are allowed to inspect the chest X-ray image [110]. Given these reasons, identifying lung lesions on chest X-ray images is an important, non-trivial task in daily, medical care. To that end, giving physicians a decision aid for this task is crucial.

Example chest X-ray images including the corresponding heatmaps are provided in Figure S3.

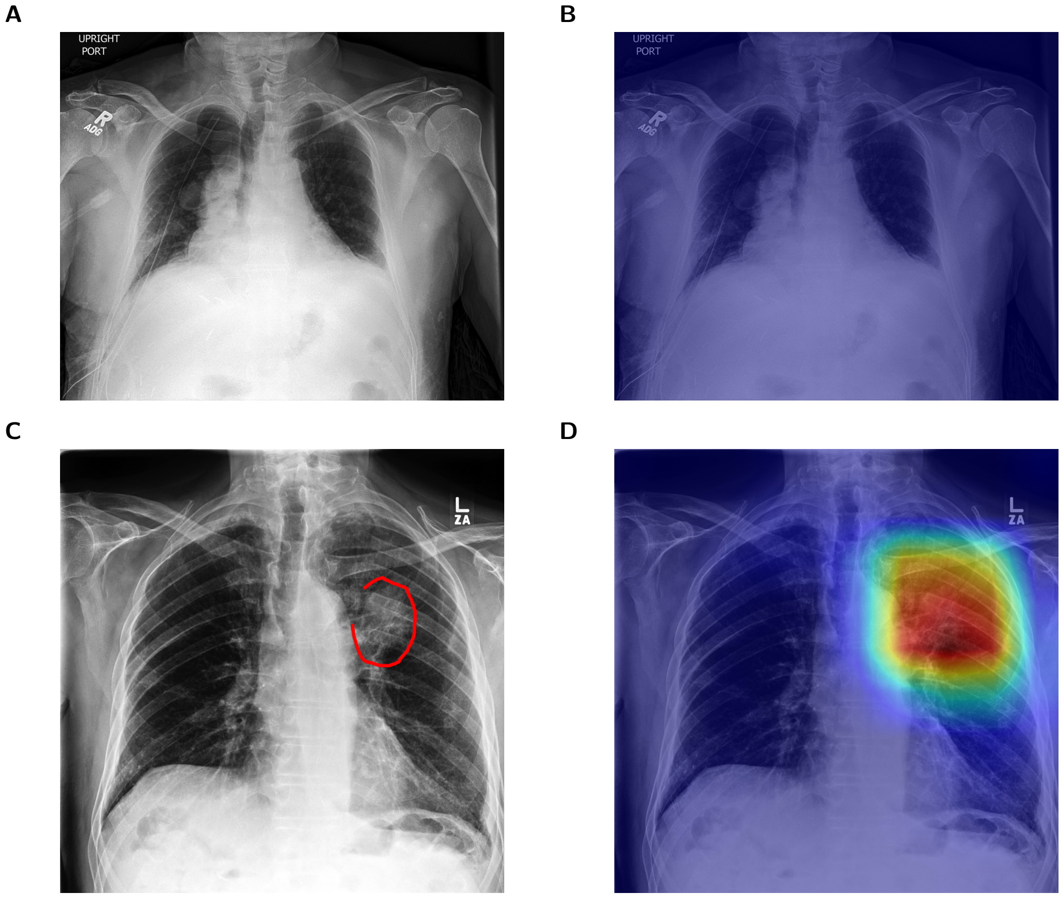

Figure S3: Example chest X-ray images with corresponding heatmaps. (**A**) Chest X-ray image without lung lesions. (**B**) Heatmap overlaid over the chest X-ray image from **A**. (**C**) Chest X-ray image with a lung lesion annotated in red by an experienced radiologist. (**D**) Heatmap overlaid over the chest X-ray image from **C**.

Supplement C Implementation of AI algorithm

C.1 Manufacturing setting

As part of this research, we implemented an AI algorithm that provided the predictions (i.e., quality scores) that were shown to the participants during the manufacturing experiment. Our AI algorithm builds upon unsupervised anomaly detection [111] and, as such, follows common standards in industry for visually analyzing the quality of product images [30, 112]. Algorithms based on unsupervised anomaly detection are particularly suitable for industrial settings because they only require a set of faultless product images to be trained. Therefore, there is no need to specify defect types beforehand, which also allows identifying quality defects that have never been observed before. This is reflected in anomaly detection where product images with sufficiently large deviations from "normal" products are labeled as defective.

AI algorithm. In our case, the AI algorithm performs unsupervised anomaly detection as follows [111]. First, we are given an existing training set T with images of faultless products. Upon deployment, an out-of-sample product image x is subject to assessment; that is, whether it is similar to any of the images $t \in T$ and thus likely faultless or whether it is highly dissimilar and thus likely defective. Here, anomaly detection compares the similarity (with regard to some similarity function d) between the new image x and the existing images $t \in T$. For each, a similarity $d(x, t)$, for all $t \in T$ is computed. If the similarity d falls below a certain threshold θ^*, an image is labeled as defective.

AI predictions. The similarity of product images is computed by following best practice in computer vision. As such, we refrain from simply computing the L2-norm (or some other norm) between x and t. The reason is that such distance would give equal weight to all pixels and cannot properly account for the semantic similarity in images. Rather, we follow established practice and compute the similarity via the so-called structural similarity index [113]. The structural similarity index is a standard computer vision method for quantifying the similarity of images between 0 (i.e., no similarity at all) and 1 (i.e., perfect similarity). Product images with a low structural similarity indicate an increased probability of a quality defect because they are less similar to the training data (i.e., images of faultless products). For details on the computation of the structural similarity index, we refer to [113]. Eventually, we scaled the structural similarity

index of all images between 0 and 100 and rounded the values to the nearest integer to enhance readability. The resulting similarity measure corresponds to the quality scores that were shown to the participants in the experiment.

Prediction performance. We evaluated the out-of-sample prediction accuracy of the AI algorithm as follows. In a first step, we mapped the quality scores onto a binary faultless/defective label. For this, we introduced a quality score of $\theta^* = 90$ as a cutoff (i.e., predicting that a product is defective if the quality score is below 90 and faultless otherwise). We then compared the predictions of the algorithm against the ground-truth quality labels provided by *Siemens*. Table S2 gives the confusion matrix for the 200 out-of-sample images used in the experiment. We measure the prediction performance via the balanced accuracy (i.e., average sensitivity across faultless and defective products). We choose the balanced accuracy as our main performance metric because it accounts for the unbalanced distribution between faultless and defective products (i.e., 172 products are faultless and 28 products are defective). The standalone AI algorithm achieves a balanced accuracy of 95.6% (i.e., $0.5 \times [169/172 + 26/28]$). Additionally, we evaluated the defect detection rate (true negative rate) of the AI algorithm, i.e., how many of the defective products were identified as such. The defect detection rate of the standalone AI algorithm was 92.9% (26/28).

Table S2: Confusion matrix comparing AI predictions with ground-truth labels in the manufacturing setting

		Predicted label	
Actual label		Faultless	Defective
	Faultless	169	3
	Defective	2	26

Explainable AI. We extended the above AI algorithm to produce explanations for each prediction as follows. We followed other research in computer vision that generates so-called "anomaly heatmaps" [30]. Anomaly heatmaps visualize in what area of an image a quality defect is predicted to be. Formally, in an anomaly heatmap, each pixel x_i is associated with a score measuring the likelihood of a defect at that location. Pixels that receive a bright color (yellow, orange, red, etc.) correspond to "anomalous" regions because they have a large distance to the training data (i.e., the pixel is dissimilar to the one in a faultless product). In contrast,

pixels that are colored in blue have a small distance to the training data and should thus be considered as "normal." For better usability, we overlay the anomaly heatmap over the actual product image (with partially transparent colors). Examples of two anomaly heatmaps are shown in Figure S4. In the experiment, the heatmaps were shown to the participants in the explainable AI treatment arm as an additional decision aid.

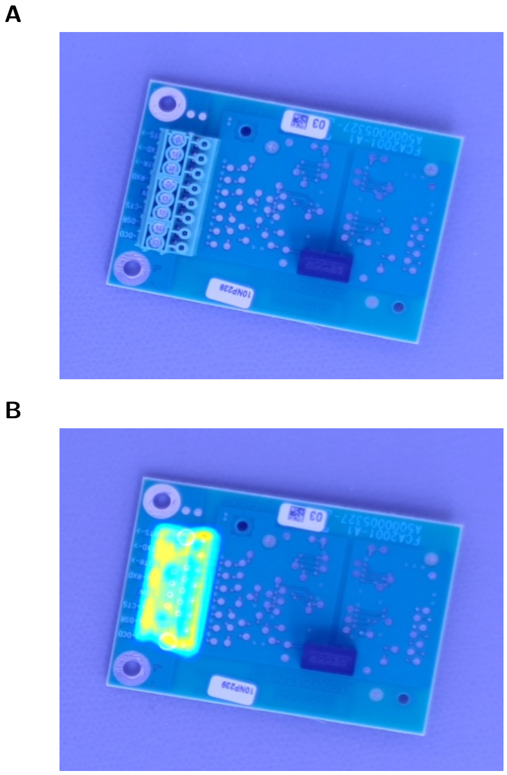

Figure S4: Anomaly heatmaps for AI predictions. (**A**) Example anomaly heatmap for a faultless product. (**B**) Example anomaly heatmap for a defective product.

C.2 Medical setting

AI algorithm. We used an already trained DenseNet121 from [36]. DenseNet121 is a convolutional neural network that is part of the DenseNet family, known for its dense connectivity pattern where each layer is connected to every other layer in a feed-forward fashion [114]. The "121" in DenseNet121 stands for the total number of layers in the network, including convolutional layers, pooling layers, and fully connected layers, summing up to 121. The DenseNet121 was set up as a multi-label classifier, which takes a chest X-ray image as input and outputs probabilities for the following 10 labels: airspace opacity, atelectasis, cardiomegaly, consolidation, edema, enlarged

cardiomediastinum, lung lesion, pleural effusion, pneumothorax, and support devices. It was trained on 224,316 chest X-ray images from 65,240 patients.

AI predictions. The probabilities returned by the DenseNet121 were mapped onto binary yes/no labels by finding the probability threshold that maximized the balanced accuracy on a validation set of chest X-ray images, which were not used during training. In order to have a score identical to the quality score from the manufacturing setting, where a smaller score indicates a greater likelihood of showing a defect and with a cutoff of 90 that divides the quality scores into defective and faultless, the following transformations were performed: (i) the probabilities were inverted, (ii) the threshold that divides the two classes was set to 90, (iii) the inverted probabilities larger than that threshold were rescaled using min-max scaling on a scale from 90 to 100, and (iv) the inverted probabilities smaller than that threshold were rescaled using min-max scaling on a scale from 0 to 90.

Prediction performance. As in the manufacturing setting, we evaluate performance of the AI algorithm on the 50 chest X-ray images by calculating the balanced accuracy and the disease detection rate. Those 50 images were neither used for training nor for finding the class dividing threshold. The standalone AI algorithm achieved a balanced accuracy of 82.2% (i.e., $0.5 \times [40/43 + 5/7]$) and a disease detection rate of 71.4% (i.e., 5/7). Additionally, the confusion matrix for the 50 images is shown in Table S3.

Table S3: Confusion matrix comparing AI predictions with ground-truth labels in the medical setting

		Predicted label	
Actual label		No lung lesion	At least one lung lesion
	No lung lesion	40	3
	At least one lung lesion	2	5

Explainable AI. As explanation technique for the above AI algorithm, we used *GradCAM* [14]. *GradCAM* outputs heatmaps similar to the anomaly heatmaps from the manufacturing setting and showed state-of-the-art localization performance on chest X-ray images across a variety of diagnoses [36]. Analogous to the manufacturing setting, pixels with bright colors (yellow, orange, red, etc.) correspond to regions that were most relevant for predicting lung lesions, whereas blue pixels were least relevant. To increase usability, heatmaps were overlaid

over the raw chest X-ray images with partially transparent colors. Examples of two heatmaps next to the original chest X-ray images are shown in Figure S3. Heatmaps were only provided to radiologists in the explainable AI treatment arm.

Supplement D Experimental interface

D.1 Manufacturing setting

The experiment was carried out via a computer interface that was analogously designed to the real-world quality inspection setup at *Siemens*. The experiment comprises the following steps: (1) the study description and study consent, (2) a tutorial on how to use the application, (3) the visual inspection task involving 200 images, (4) a post-experimental questionnaire.

Depending on the randomly assigned treatment, different versions of the quality inspection interface were shown to participants (Figure S5). Similar to the real-world setting at *Siemens*, all participants had access to a reference image, which showed a faultless product. The participants were asked to evaluate each of the 200 images individually and to make an "approve" (faultless product) or "reject" (defective product) decision by clicking the respective buttons. This represents the quality assessments that we use for all analyses. The participants were allowed to change their quality assessment before submitting their decision and proceeding to the next image. Once a decision was submitted, participants could no longer return to the previous image. Overall, the participants were given 35 minutes to solve the task, which corresponds to realistic field conditions. The remaining time was always shown on the top of the interface.

We tracked several metrics during the experiment. In the tutorial, we tracked whether participants were following the steps correctly and screened out those that did not complete the tutorial successfully. During the visual inspection task, we tracked the final quality assessment (i.e., faultless or defective) and the decision speed of the users. In the post-experimental questionnaire, we saved the answers to individual questions. The aggregated user data were stored in a database and later converted into a comma-separated values (CSV) file.

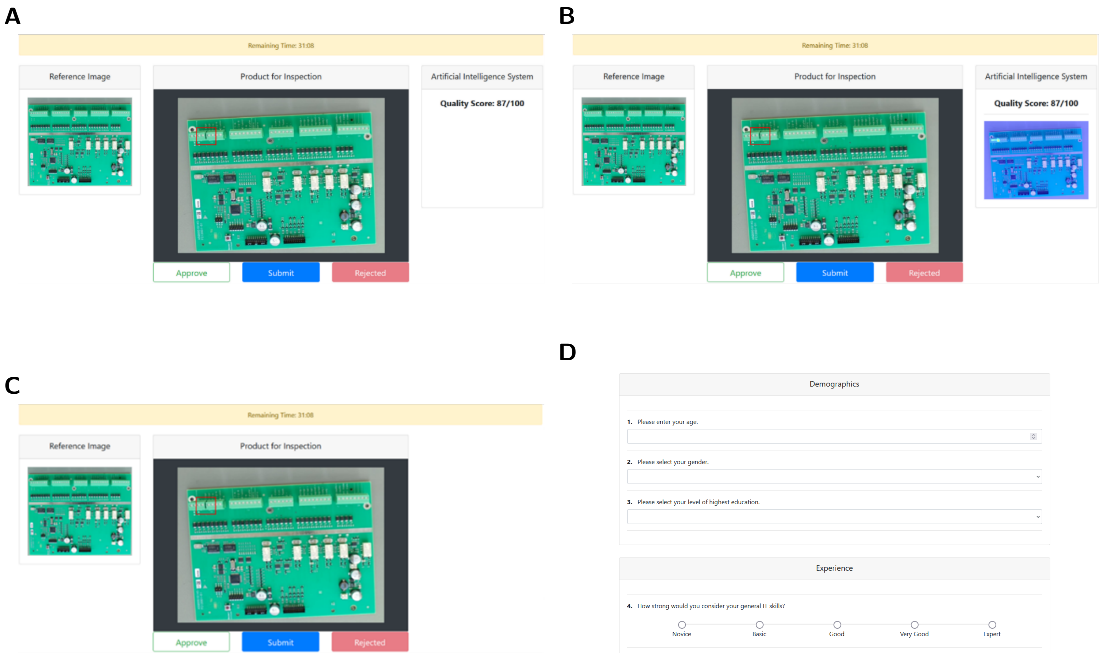

Figure S5: Different interfaces depending on treatment arm. (**A**) The interface for the black-box AI. (**B**) The interface for the explainble AI. (**C**) The interface for the human without AI treatment. (**D**) The interface for the post-experimental questionnaire.

D.2 Medical setting

The experiment was conducted via Qualtrics. The experiment was divided in the following steps: (1) physician confirmation and study consent, (2) a tutorial on how to perform the experiment, (3) the visual inspection task consisting of 50 chest X-ray images, and (4) a post-experimental questionnaire.

A different version of the chest X-ray inspection interface was shown to radiologists depending on the randomly assigned treatment (Figure S6). For both treatment arms, the chest X-ray image to inspect was shown on the left. To the right of it, an enlarged view was shown, which could be altered by moving the mouse over the chest X-ray image. Radiologists in the treatment arm with explainable AI additionally received a heatmap, which was displayed right of the enlarged view. The radiologists were asked to inspect 50 chest X-ray images and to answer the question "Is at least one lung lesion visible in the chest X-ray image below?" with either "YES" or "NO" for each image. The radiologists were allowed to change their assessment before submitting their decision (clicking the blue arrow button to proceed to the next page). Each chest X-ray image was shown on a separate page and radiologists were not allowed to go back to a previous image

once a decision was submitted. The radiologists had 35 minutes to complete the task and the remaining time was always shown on the top-left of the page.

Several metrics were recorded during the experiment: In the tutorial, we tracked whether radiologists understood how to perform the task. In the visual inspection task, the final assessment for each image as well as the corresponding decision speed were recorded. In the post-hoc questionnaire, we saved the answers to the individual questions. The data was stored on Qualtrics and exported as a CSV file.

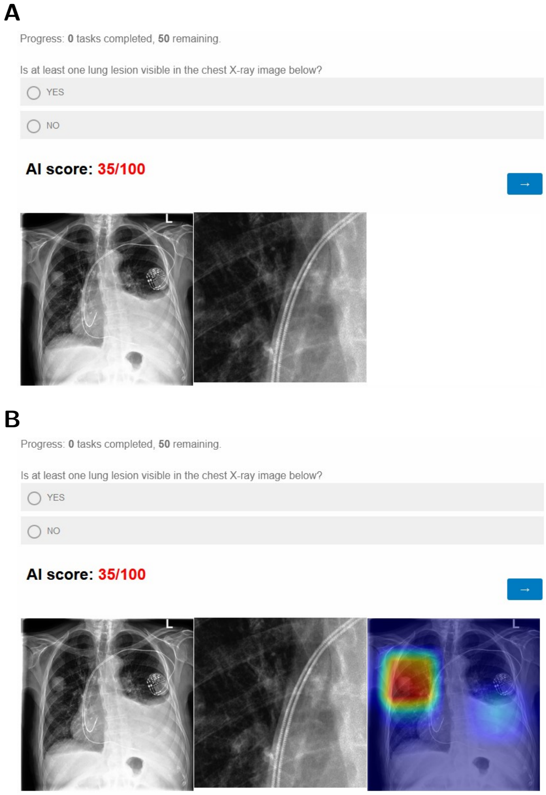

Figure S6: Different interfaces depending on treatment arm. (**A**) The interface for the black-box AI. (**B**) The interface for the explainble AI.

Supplement E Randomization checks

E.1 Study 1: Manufacturing experiment

We performed randomization checks to confirm that the distribution of workers in the two treatment arms of the manufacturing experiment was unbiased. The following demographic variables were collected: age bracket [<20, 20–30, 30–40, 40–50, 50–60, 60–70, >70], gender [male, female, not listed], and highest level of education [ISCED1, ISCED2, ISCED3, ISCED4, ISCED5, ISCED6, ISCED7].[2] We further collected the participant-specific tenure at *Siemens* measured in years since start of employment. Table S4 reports the observed frequencies and the mean tenure (with standard deviation in parentheses) for both treatment arms. The randomization checks for age, gender, and education are based on $\mathcal{X}^2$-tests of independence. The randomization check for tenure is based on a two-sided Welch's t-test. The results suggest no statistically significant differences between the participants in the two treatment arms.

Table S4: Randomization checks for manufacturing experiment

	**Human with black-box AI**	**Human with explainable AI**	***P*-value**
Age	0 | 1 | 7 | 8 | 4 | 2 | 0	0 | 1 | 3 | 9 | 12 | 1 | 0	0.223
Gender	16 | 6 | 0	17 | 9 | 0	0.815
Education	0 | 9 | 4 | 2 | 3 | 3 | 1	0 | 14 | 2 | 3 | 3 | 3 | 1	0.897
Tenure	11.91 (8.83)	15.38 (10.42)	0.217
Observations	22	26	–

Notes: The table reports the frequency of participants that fall in the specific subgroups of age, gender, and education (separated by vertical bars) and the average tenure per treatment arm (standard deviation in parentheses). The P-values for the randomization checks are computed based on $\mathcal{X}^2$-tests of independence (age, gender, education) and a two-sided Welch's t-test (tenure).

E.2 Study 2: Medical experiment

We performed a randomization check to confirm that the distribution of radiologists with respect to tenure in the two treatment arms of the medical experiment was unbiased. The randomization check is based on a two-sided Welch's t-test. The result suggests no statistically significant differences between the radiologists in the two treatment arms.

[2]UNESCO. *International Standard Classification of Education (ISCED)*. URL: http://uis.unesco.org/en/topic/international-standard-classification-education-isced, last accessed on November 18, 2024.

Table S5: Randomization checks for medical experiment

	**Human with black-box AI**	**Human with explainable AI**	***P*-value**
Tenure	11.89 (8.96)	15.4 (11.71)	0.08
Observations	61	52	–

Notes: The table reports the average tenure per treatment arm (standard deviation in parentheses). The P-value is computed based on a two-sided Welch's t-test.

Supplement F Robustness of the heatmap

We applied two additional algorithms to generate the heatmaps in the medical setting in order to show that different algorithms lead to similar heatmaps. In particular, we compared our heatmaps generated by *GradCAM* to heatmaps generated by *DeepLIFT* and *LRP* (for an introduction to these two methods see Supplement A) [25, 23]. Figure S7 shows the heatmaps generated by the three distinct algorithms for the eight chest X-ray images, where the AI algorithm predicted that lung lesions are visible.

Additionally, we calculated Pearson correlation coefficients between the heatmaps generated by different algorithms to quantify whether they highlight similar regions. The Pearson correlation coefficient is calculated via $r = \frac{\mathrm{cov}(x_i, x_j)}{\sqrt{\mathrm{Var}(x_i)\,\mathrm{Var}(x_j)}}$, where x_i and x_j denote the flattened array of heatmaps generated by algorithm i and j. The average Pearson correlation coefficient between *GradCAM* and *DeepLIFT* is $r = 0.92$, and the average Pearson correlation coefficient between *GradCAM* and *LRP* is $r = 0.63$. The exact Pearson correlation coefficient for each heatmap pair is reported in Figure S7. The Pearson correlation coefficient was statistically significant for each pair of heatmaps ($P < 0.001$). In general, we observe that all three algorithms produce similar heatmaps. The heatmaps we used in our medical setting (generated by *GradCAM*) are more similar to the heatmaps generated by *DeepLIFT* than to those generated by *LRP*.

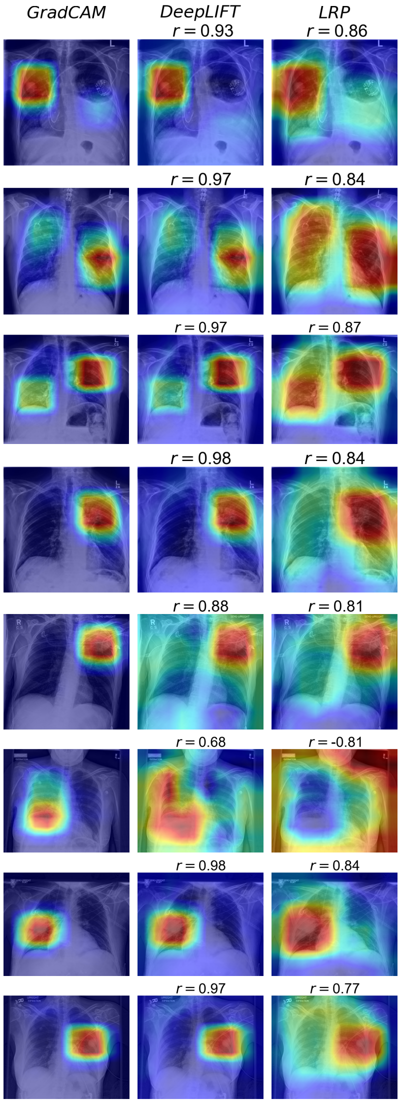

Figure S7: Heatmaps generated by three different algorithms. The left column shows the heatmaps generated by *GradCAM* (the algorithm we used for our medical experiment). The middle columns shows the heatmaps generated by *DeepLIFT*. The right column shows the heatmaps generated by *LRP*. r denotes the Pearson correlation coefficient between the heatmaps generated by *GradCAM* (the algorithm we used) and *DeepLIFT*/*LRP*, respectively.

Supplement G Results with precision as task performance metric

In addition to the balanced accuracy and defect detection rate, we also report precision as a metric for task performance of the participants in combination with the defect/disease detection rate. Formally, precision is computed via TN/PN with true negatives TN and predicted negatives PN. We again compare the effect of augmenting humans with explainable AI versus black-box AI. Figure S8 reports the results for the manufacturing experiment (Study 1) and Figure S9 for the medical experiment (Study 2)

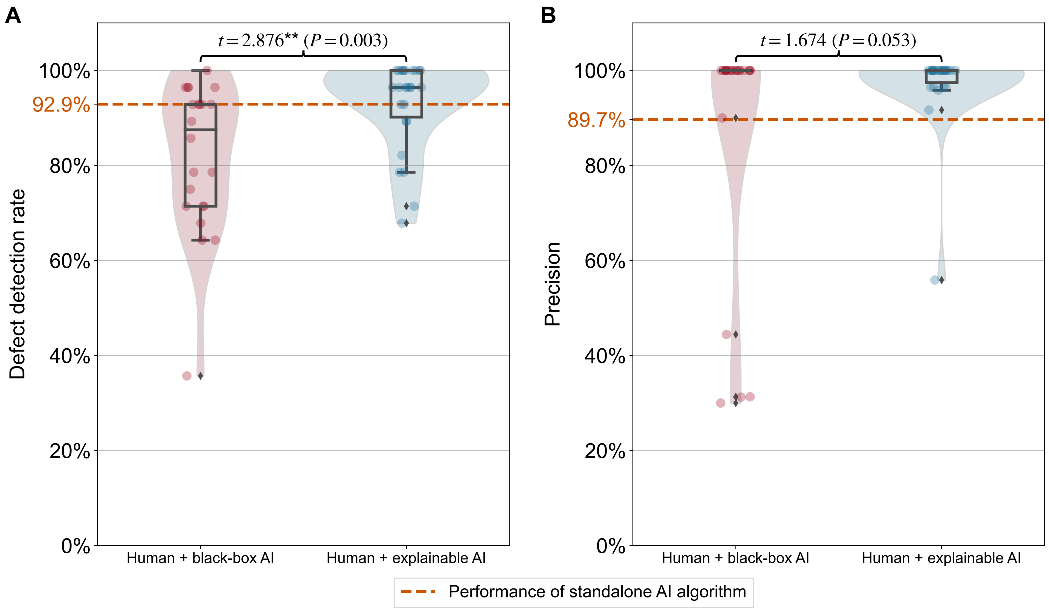

Figure S8: Results of manufacturing experiment. The boxplots compare the task performance between the two treatments: black-box AI and explainable AI. The task performance is measured by the defect detection rate (**A**) and the precision (**B**) based on the quality assessment of workers and the ground-truth labels of the product images. The standalone AI algorithm attains a defect detection rate of 92.9% and a precision of 89.7% (orange dashed lines). Statistical significance is based on a one-sided Welch's t-test ($^{***}P < 0.001$, $^{**}P < 0.01$, $^{*}P < 0.05$). In the boxplots, the center line denotes the median; box limits are upper and lower quartiles; whiskers are defined as the 1.5x interquartile range.

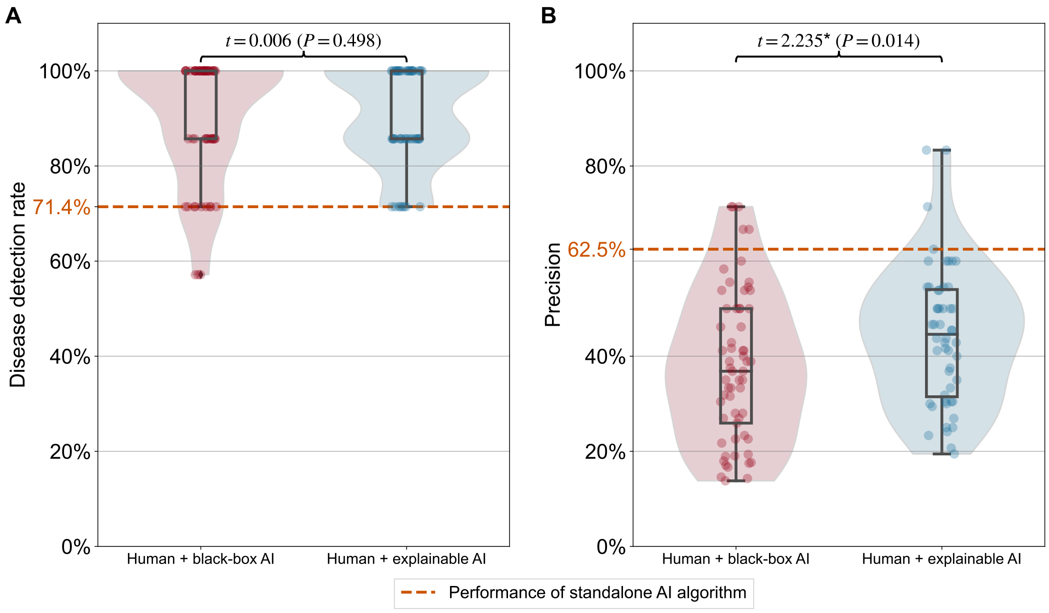

Figure S9: Results of medical experiment. The boxplots compare the task performance between the two treatments: black-box AI and explainable AI. The task performance is measured by the disease detection rate (**A**) and the precision (**B**) based on the quality assessment of radiologists and the ground-truth labels of the chest X-ray images. The standalone AI algorithm attains a disease detection rate of 71.4% and a precision of 62.5% (orange dashed lines). Statistical significance is based on a one-sided Welch's t-test ($^{***}P < 0.001$, $^{**}P < 0.01$, $^{*}P < 0.05$). In the boxplots, the center line denotes the median; box limits are upper and lower quartiles; whiskers are defined as the 1.5x interquartile range.

Supplement H Regression models

This section reports various regression models estimating the treatment effect of augmenting humans with explainable AI. The models are estimated via

$$Y_i = \beta_0 + \beta_1\, Treatment_i + \beta_2\, X_i + \varepsilon_i, \qquad \text{(S1)}$$

where Y_i is the observed task performance (i.e., balanced accuracy or defect/disease detection rate), $Treatment_i$ is a binary variable which equals 0 if participant i received the black-box AI treatment and 1 if participant i received the explainable AI treatment, and X_i is the vector of participant-specific control variables. The above regression is estimated via ordinary least squares (OLS).

We acknowledge that the balanced accuracy is only defined between 0 and 100. Because OLS regression models could return values below 0 and above 100, we additionally estimate quasi-binomial regression models with a logit link function. For this, we set the scale parameter of the regression models to the Pearson $\mathcal{X}^2$-statistic divided by the residual degrees of freedom.

H.1 Study 1: Manufacturing experiment

Table S6 reports three OLS regression models estimating the treatment effect with different control variables. Model (1) estimates the treatment effect for explainable AI with demographic controls (age, gender, and highest level of education) and the tenure at *Siemens* measured in years from start of employment. Model (2) estimates the treatment effect for explainable AI with demographic controls, tenure, and self-reported IT skills (ranging from 1: "novice" to 5: "expert"). Model (3) estimates the treatment effect for explainable AI with demographic controls, tenure, self-reported IT skills, and the decision speed (median across the 200 images). All three models return a significant treatment effect for both metrics (balanced accuracy and defect detection rate) as dependent variables.

Table S6: OLS regression results for treatment effect (manufacturing experiment)

	Balanced accuracy			Defect detection rate		
	**Model (1)**	**Model (2)**	**Model (3)**	**Model (1)**	**Model (2)**	**Model (3)**
Treatment	8.131***	7.513***	7.508**	11.783**	10.914**	10.888**
(explainable AI)	(2.087)	(2.098)	(2.117)	(3.732)	(3.790)	(3.717)
Demographics	Yes	Yes	Yes	Yes	Yes	Yes
Tenure	Yes	Yes	Yes	Yes	Yes	Yes
IT skills	No	Yes	Yes	No	Yes	Yes
Decision speed	No	No	Yes	No	No	Yes
Observations	48	48	48	48	48	48

Notes: The table reports three OLS regression models with different sets of control variables and two different metrics as dependent variables. The standard errors of the treatment effect are reported in parentheses. Statistical significance: $^{***}P < 0.001$, $^{**}P < 0.01$, $^{*}P < 0.05$.

Table S7 reports three quasi-binomial regression models estimating the treatment effect with the same control variables as before. Again, all three models return a significant treatment effect for both metrics as dependent variables.

Table S7: Quasi-binomial regression results for treatment effect (manufacturing experiment)

	Balanced accuracy			Defect detection rate		
	**Model (1)**	**Model (2)**	**Model (3)**	**Model (1)**	**Model (2)**	**Model (3)**
Treatment	1.295***	1.175**	1.184***	1.157**	1.060**	1.089**
(explainable AI)	(0.340)	(0.358)	(0.357)	(0.369)	(0.386)	(0.375)
Demographics	Yes	Yes	Yes	Yes	Yes	Yes
Tenure	Yes	Yes	Yes	Yes	Yes	Yes
IT skills	No	Yes	Yes	No	Yes	Yes
Decision speed	No	No	Yes	No	No	Yes
Observations	48	48	48	48	48	48

Notes: The table reports three quasi-binomial regression models with different sets of control variables and two different metrics as dependent variables. The standard errors of the treatment effect are reported in parentheses. Statistical significance: $^{***}P < 0.001$, $^{**}P < 0.01$, $^{*}P < 0.05$.

H.2 Study 2: Medical experiment

Table S8 reports three OLS regression models estimating the treatment effect with different control variables. Model (1) estimates the treatment effect for explainable AI with tenure measured in years as a control variable. Model (2) estimates the treatment effect for explainable AI with tenure and self-reported IT skills (ranging from 1: "novice" to 5: "expert"). Model (3) estimates the treatment effect for explainable AI with tenure, self-reported IT skills, and the

decision speed (median across the 50 images). All three models return a significant treatment effect for balanced accuracy as a dependent variable.

Table S8: OLS regression results for treatment effect (medical experiment)

	Balanced accuracy			Defect detection rate		
	**Model (1)**	**Model (2)**	**Model (3)**	**Model (1)**	**Model (2)**	**Model (3)**
Treatment	4.637*	4.452*	4.473*	0.129	0.304	0.645
(explainable AI)	(1.834)	(1.853)	(1.863)	(2.286)	(2.312)	(2.215)
Tenure	Yes	Yes	Yes	Yes	Yes	Yes
IT skills	No	Yes	Yes	No	Yes	Yes
Decision speed	No	No	Yes	No	No	Yes
Observations	113	113	113	113	113	113

Notes: The table reports three OLS regression models with different sets of control variables and two different metrics as dependent variables. The standard errors of the treatment effect are reported in parentheses. Statistical significance: $^{***}P < 0.001$, $^{**}P < 0.01$, $^{*}P < 0.05$.

Table S9 reports three quasi-binomial regression models estimating the treatment effect with the same control variables as before. Again, all three models return a significant treatment effect for balanced accuracy as dependent variable.

Table S9: Quasi-binomial regression results for treatment effect (medical experiment)

	Balanced accuracy			Defect detection rate		
	**Model (1)**	**Model (2)**	**Model (3)**	**Model (1)**	**Model (2)**	**Model (3)**
Treatment	0.308*	0.296*	0.297*	0.015	0.035	0.073
(explainable AI)	(0.120)	(0.121)	(0.122)	(0.264)	(0.268)	(0.259)
Tenure	Yes	Yes	Yes	Yes	Yes	Yes
IT skills	No	Yes	Yes	No	Yes	Yes
Decision speed	No	No	Yes	No	No	Yes
Observations	113	113	113	113	113	113

Notes: The table reports three quasi-binomial regression models with different sets of control variables and two different metrics as dependent variables. The standard errors of the treatment effect are reported in parentheses. Statistical significance: $^{***}P < 0.001$, $^{**}P < 0.01$, $^{*}P < 0.05$.

Supplement I Analysis with excluded participants

In our data analyses, we followed our preregistration and excluded participants who did not finish the task in time or participants with obvious misbehavior. Specifically, six and two participants were excluded from Study 1 and Study 2, respectively, because they did not finish the task in time. Further, in Study 1, we excluded participants who did not label a single product as defective (which corresponds to one participant) and in Study 2, radiologists were excluded if they assigned only one label to all chest X-ray images (which corresponds to 1 radiologist). Further, participants whose performance was more than three standard deviations worse than the mean of their respective treatment arm were excluded (which corresponds to one worker in Study 1 and two radiologists in Study 2). This section repeats the OLS regression from the main paper (without control variables) with participants who were excluded due to obvious misbehavior. Overall, we arrive at consistent findings.

Table S10: Excluded participants across treatment arms

	**Study 1: Manufacturing**		**Study 2: Medical**	
	Black-box AI	Explainable AI	Black-box AI	Explainable AI
Time-out	5	1	0	2
No defective	1	0	–	–
Single label	–	–	1	0
Worse than 3σ	0	1	1	1

I.1 Study 1: Manufacturing experiment

Table S11 reports the OLS regression model estimating the treatment effect for all different combinations of exclusion criteria. As in our main analysis, the effect of explainable AI is statistically significant for both metrics, balanced accuracy and defect detection rate. The only exception is for the defect detection rate when workers that timed-out and did not label a single product as defective were excluded while including those that were worse than three standard deviations than the mean.

Table S11: OLS regression results with excluded participants (manufacturing experiment)

Excluded:			Observations	Balanced accuracy	Defect detection rate
Time-out	No defective	Worse than 3σ			
✗	✗	✗	56	8.143** (2.754)	11.590* (4.965)
✓	✗	✗	50	7.944* (3.016)	12.192* (5.472)
✗	✓	✗	55	6.769** (2.432)	8.651* (4.077)
✗	✗	✓	54	8.120*** (2.042)	10.961** (3.391)
✓	✓	✗	49	6.253* (2.642)	8.628 (4.477)
✓	✗	✓	48	7.653** (2.178)	11.014** (3.680)
✗	✓	✓	54	8.120*** (2.042)	10.961** (3.391)

Notes: The table reports the OLS regression model with two different metrics as dependent variables for all combinations of exclusion criteria. The standard errors of the treatment effect are reported in parentheses. Statistical significance: $^{***}P < 0.001$, $^{**}P < 0.01$, $^{*}P < 0.05$.

I.2 Study 2: Medical experiment

Table S12 reports the OLS regression model estimating the treatment effect for all different combinations of exclusion criteria. As in our main analysis, the effect of explainable AI is statistically significant for balanced accuracy, irrespective of the exclusion criteria. Only when radiologists that assigned one label to all chest X-ray images are excluded the treatment effect for balanced accuracy was not statistically significant. For the disease detection rate, the treatment effect was not statistically significant in the main analysis. This did not change when different exclusion criteria are considered.

Table S12: OLS regression results with excluded participants (medical experiment)

Excluded:			Observations	Balanced accuracy	Disease detection rate
Time-out	Single label	Worse than 3σ			
✗	✗	✗	118	4.418* (2.067)	-0.282 (2.337)
✓	✗	✗	116	5.266** (1.996)	0.543 (2.274)
✗	✓	✗	117	3.967 (2.026)	-0.121 (2.349)
✗	✗	✓	115	4.988** (1.846)	-0.256 (2.212)
✓	✓	✗	115	4.815* (1.950)	0.704 (2.286)
✓	✗	✓	114	5.162** (1.857)	-0.168 (2.232)
✗	✓	✓	114	4.520* (1.790)	-0.102 (2.224)

Notes: The table reports the OLS regression model with two different metrics as dependent variables for all combinations of exclusion criteria. The standard errors of the treatment effect are reported in parentheses. Statistical significance: $^{***}P < 0.001$, $^{**}P < 0.01$, $^{*}P < 0.05$.

Supplement J Experiment with non-experts

To extend our findings to non-experts, we conducted a third experiment with participants recruited via Amazon MTurk to perform the visual inspection task in the manufacturing setting. We chose the manufacturing task for this because, in principle, non-experts could compare electronic products against a reference image (a faultless product looks always identical). For chest X-ray images, this is hardly possible since these can look very different across different healthy patients. We followed common practice by only admitting MTurk workers with an approval rating above 95% [115]. We prevented double participation by tracking the IP of participants. The participants received a base compensation ($5) and had the opportunity to earn a performance-dependent bonus proportional to the correctly labeled quality defects ($3). Participants were randomly assigned to one of the following three treatments: (a) human with black-box AI, (b) human with explainable AI, and (c) human without AI. Following the preregistration, we aimed to include approximately 600 participants excluding dropouts. We thus recruited 861 participants (U.S. residents) who started the study between July 19 and July 21, 2021. Out of them, 117 participants did not complete the study; 152 failed the tutorial; 92 did not finish on time; and 70 participants were excluded due to obvious misbehavior. The final sample consisted of 430 participants, out of which 288 were assigned to treatment arms (a) or (b), performing $N = 57,600$ assessments of electronic products.

We found that participants supported by explainable AI reached a higher task performance than the participants supported by black-box AI across both metrics (Figure S10). Participants with black-box AI treatment only achieved a balanced accuracy with a mean of 81.4%, whereas participants with explainable AI treatment achieved a balanced accuracy with a mean of 87.6%. We then estimated the overall treatment effect on the task performance by regressing the balanced accuracy on the treatment (black-box AI = 0, explainable AI = 1). The regression results suggest that the treatment effect of explainable AI is statistically significant and large ($\beta = 6.252$, $SE = 1.733$, $t = 3.608$, $P < 0.001$, 95 % CI = $[2.841, 9.664]$); that is, an improvement of 6.3 percentage points. Accordingly, participants equipped with explainable AI achieved a higher defect detection rate with mean of 77.7% compared to participants with black-box AI with a mean of 66.4%. Again, the regression results showed a large and statistically significant treatment effect of explainable AI ($\beta = 11.271$, $SE = 3.276$, $t = 3.440$, $P = 0.001$, 95 % CI = $[4.822, 17.720]$).

The regression results remain statistically significant for both metrics when including relevant control variables (demographics, self-reported IT skills, and decision speed) in the regression model (Supplement J.3).

We additionally compared how humans without AI support performed relative to humans with black-box AI or explainable AI. For this, we further recruited 142 participants and assigned them to a third treatment: human without AI. Here, participants only got images of the to-be-inspected products and the corresponding reference images of faultless products, but not the AI-based quality scores or the heatmaps. We found that participants without AI support only achieved a balanced accuracy with a mean of 72.4% (Figure S10) and were significantly outperformed by participants with both black-box AI ($t = 5.507$, $P < 0.001$) and explainable AI ($t = 9.017$, $P < 0.001$). Similar results were found for the defect detection rate, where participants without AI achived a mean of 53.6% and were outperformed by participants with both black-box AI ($t = 5.202$, $P < 0.001$) and explainable AI ($t = 8.733$, $P < 0.001$).

We further explored whether the performance difference between the treatments (black-box AI versus explainable AI) was associated with adherence to AI predictions. For this, we compared how likely participants were to follow quality scores that were accurate (i.e., the AI prediction for the inspected product was correct). The results suggest that participants with explainable AI were more likely to adhere to accurate quality scores than participants with black-box AI (mean = 92.9% for black-box AI, mean = 95.2% for explainable AI). Overall, participants supported by black-box AI were 47.9% more likely to erroneously overrule an AI prediction, despite the prediction being accurate ($t = 2.377$, $P = 0.009$). We also analyzed whether participants were able to identify and overrule AI predictions that were wrong. Here, we found that participants supported by black-box AI only overruled 65.8% of the wrong AI predictions, whereas participants supported by explainable AI overruled 79.1% of the wrong AI predictions. The difference between both treatments is statistically significant ($t = 4.563$, $P < 0.001$). Evidently, explainable AI gives a powerful decision aid: it made participants not only less averse to following accurate AI predictions but also helped them overrule wrong AI predictions.

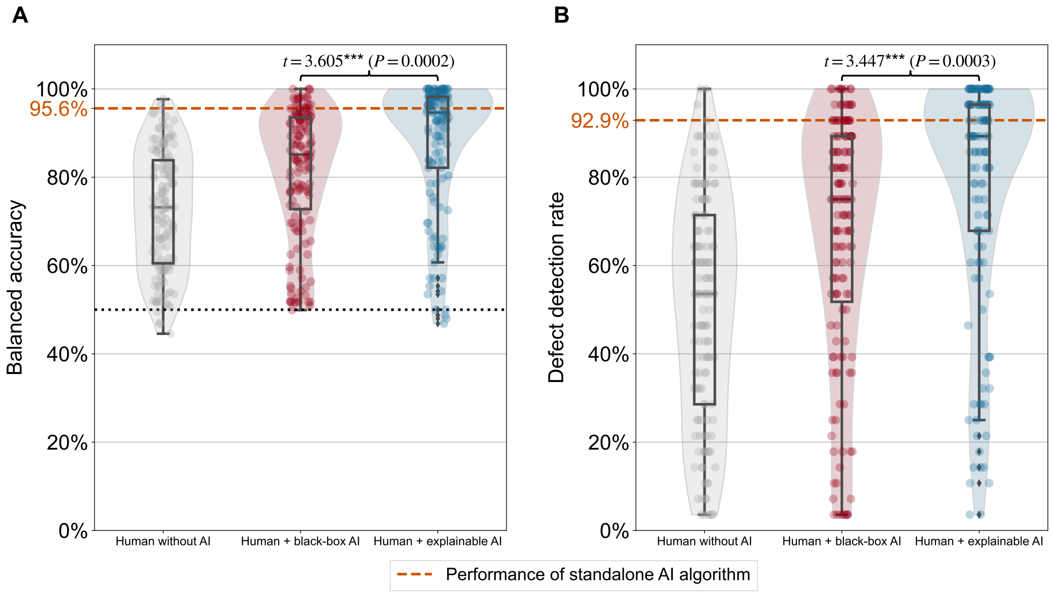

Figure S10: Results of non-experts experiment. The boxplots compare the task performance between humans without AI, with black-box AI, and with explainable AI. The task performance is measured by the balanced accuracy (**A**) and the defect detection rate (**B**) based on the quality assessment of participants and the ground-truth labels of the product images. A balanced accuracy of 50% provides a naïve baseline corresponding to a random guess (black dotted line). The standalone AI algorithm attains a balanced accuracy of 95.6% and a defect detection rate of 92.9% (orange dashed lines). Statistical significance is based on a one-sided Welch's t-test ($^{***}P < 0.001$, $^{**}P < 0.01$, $^{*}P < 0.05$). In the boxplots, the center line denotes the median; box limits are upper and lower quartiles; whiskers are defined as the 1.5x interquartile range.

We also assessed whether participants with explainable AI invested more time for the visual inspection task. For this, we compared participants' median decision speeds across the 200 product images. No significant differences ($t = 0.584$, $P = 0.280$) between both treatments (mean = 4.61 s for black-box AI, mean = 4.50 s for explainable AI) were observed. Hence, explainable AI improved task performance, but not at the cost of decision speed.

J.1 Results with precision as task performance metric

In Figure S11, the results with precision as task performance metric are shown. We find that non-experts augmented by explainable AI are more precise in identifying defective electronic products in comparison to peers supported by black-box AI.

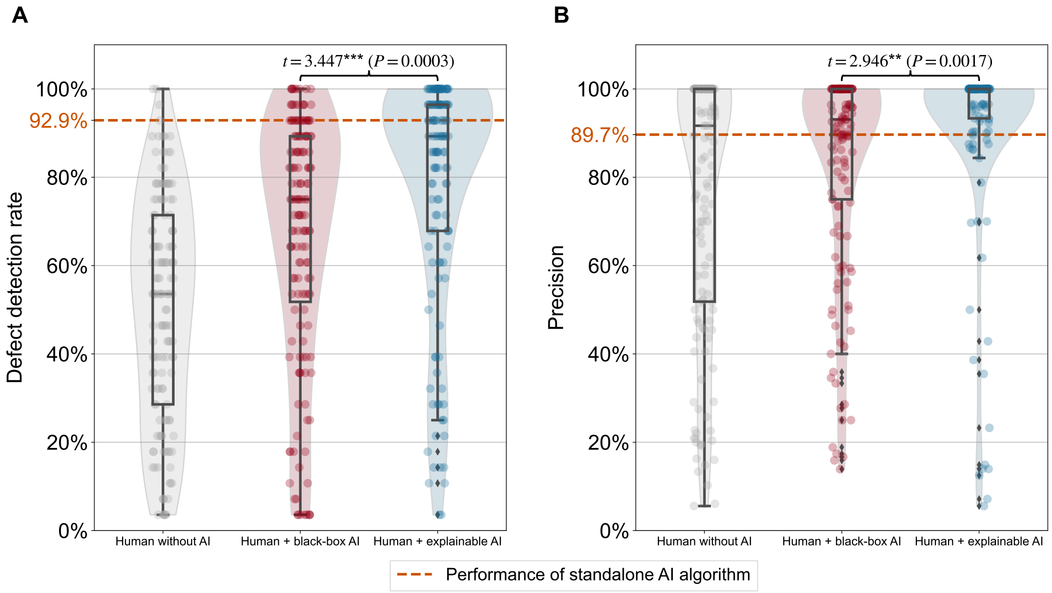

Figure S11: Results of non-expert experiment. The boxplots compare the task performance between the two treatments: black-box AI and explainable AI. The task performance is measured by the defect detection rate (**A**) and the precision (**B**) based on the quality assessment of radiologists and the ground-truth labels of the chest X-ray images. The standalone AI algorithm attains a defect detection rate of 92.9% and a precision of 89.7% (orange dashed lines). Statistical significance is based on a one-sided Welch's t-test ($^{***}P < 0.001$, $^{**}P < 0.01$, $^{*}P < 0.05$). In the boxplots, the center line denotes the median; box limits are upper and lower quartiles; whiskers are defined as the 1.5x interquartile range.

J.2 Randomization checks

We performed randomization checks to confirm that the distribution of participants in the three treatment arms of the non-experts experiment was unbiased. The following demographic variables were collected: age bracket [<20, 20–30, 30–40, 40–50, 50–60, 60–70, >70], gender [male, female, not listed], and highest level of education [no schooling, primary school, some high-school; no degree, high school degree, Bachelor's degree, Master's degree, doctorate]. Table S13 reports the observed frequencies in the three treatment arms. The randomization checks are based on $\mathcal{X}^2$-tests of independence. The results suggest no statistically significant differences between the participants in the three treatment arms.

Table S13: Randomization checks for non-experts experiment

	Human with black-box AI	Human with explainable AI	Human without AI	*P*-value
Age	0 | 39 | 58 | 36 | 16 | 1 | 1	0 | 26 | 56 | 34 | 13 | 8 | 0	1 | 46 | 49 | 28 | 13 | 5 | 0	0.168
Gender	104 | 47 | 0	86 | 51 | 0	89 | 53 | 0	0.443
Education	0 | 0 | 1 | 30 | 88 | 32 | 0	0 | 0 | 1 | 24 | 91 | 21 | 0	0 | 0 | 2 | 16 | 90 | 34 | 0	0.278
Observations	151	137	142	–

Notes: The table reports the frequency of participants that fall in the specific subgroups of age, gender, and education (separated by vertical bars). The P-values for the randomization checks are computed based on $\mathcal{X}^2$-tests of independence.

J.3 Regression models

Table S14 reports three OLS regression models estimating the treatment effect with different control variables. Model (1) estimates the treatment effect for explainable AI with demographic controls (age, gender, and highest level of education). Model (2) estimates the treatment effect for explainable AI with demographic controls and self-reported IT skills (ranging from 1: "novice" to 5: "expert"). Model (3) estimates the treatment effect for explainable AI with demographic controls, self-reported IT skills, and the decision speed (median across the 200 images). All three models return a significant treatment effect for both metrics (balanced accuracy and defect detection rate) as dependent variables.

Table S14: OLS regression results for treatment effect (non-experts experiment)

	Balanced accuracy			Defect detection rate		
	Model (1)	Model (2)	Model (3)	Model (1)	Model (2)	Model (3)
Treatment (explainable AI)	5.792*** (1.729)	5.832*** (1.720)	5.570** (1.707)	10.435** (3.274)	10.509** (3.258)	10.299** (3.263)
Demographics	Yes	Yes	Yes	Yes	Yes	Yes
IT skills	No	Yes	Yes	No	Yes	Yes
Decision speed	No	No	Yes	No	No	Yes
Observations	288	288	288	288	288	288

Notes: The table reports three OLS regression models with different sets of control variables and two different metrics as dependent variables. The standard errors of the treatment effect are reported in parentheses. Statistical significance: $^{***}P < 0.001$, $^{**}P < 0.01$, $^{*}P < 0.05$.

Table S15 reports three quasi-binomial regression models estimating the treatment effect with the same control variables as before. Again, all three models return a significant treatment effect

for both metrics as dependent variables.

Table S15: Quasi-binomial regression results for treatment effect (non-experts experiment)

	Balanced accuracy			Defect detection rate		
	**Model (1)**	**Model (2)**	**Model (3)**	**Model (1)**	**Model (2)**	**Model (3)**
Treatment	0.449**	0.455***	0.442**	0.528**	0.536**	0.528**
(explainable AI)	(0.137)	(0.136)	(0.136)	(0.168)	(0.167)	(0.168)
Demographics	Yes	Yes	Yes	Yes	Yes	Yes
IT skills	No	Yes	Yes	No	Yes	Yes
Decision speed	No	No	Yes	No	No	Yes
Observations	288	288	288	288	288	288

Notes: The table reports three quasi-binomial regression models with different sets of control variables and two different metrics as dependent variables. The standard errors of the treatment effect are reported in parentheses. Statistical significance: $^{***}P < 0.001$, $^{**}P < 0.01$, $^{*}P < 0.05$.

J.4 Analysis with excluded participants

Table S16 reports the number of patients that were excluded according to the three different criteria that we have preregistered.

Table S16: Excluded participants across treatment arms

	Black-box AI	Explain-able AI	Without AI
Time-out	26	31	35
No defective	21	21	27
Worse than 3σ	0	1	0

Table S17 reports the OLS regression model estimating the treatment effect for all different combinations of exclusion criteria. As in our main analysis, the effect of explainable AI is statistically significant for both metrics, balanced accuracy and defect detection rate, irrespective of the exclusion criteria.

Table S17: OLS regression results with excluded participants (non-experts experiment)

Excluded:			Observations	Balanced accuracy	Defect detection rate
Time-out	No defective	Worse than 3σ			
✗	✗	✗	388	6.344*** (1.825)	11.539** (3.548)
✓	✗	✗	331	4.843* (1.990)	8.765* (3.902)
✗	✓	✗	342	6.966*** (1.648)	12.652*** (3.023)
✗	✗	✓	387	6.561*** (1.817)	11.788*** (3.549)
✓	✓	✗	289	5.918*** (1.756)	10.863** (3.288)
✓	✗	✓	330	5.102* (1.980)	9.054* (3.904)
✗	✓	✓	341	7.238*** (1.631)	12.988*** (3.014)

Notes: The table reports the OLS regression model with two different metrics as dependent variables for all combinations of exclusion criteria. The standard errors of the treatment effect are reported in parentheses. Statistical significance: $^{***}P < 0.001$, $^{**}P < 0.01$, $^{*}P < 0.05$.

Supplement K Preregistered hypotheses

The following hypotheses were preregistered at https://osf.io/7djxb (Study 1) and https://osf.io/69yqt (Study 2):

Hypothesis 1 (H1): *Explainable AI improves the overall decision performance (measured by the balanced accuracy and defect detection rate) compared to humans without AI (i.e., manual inspection) ($\alpha = 0.05$).*

Hypothesis 2 (H2): *Explainable AI improves the overall decision performance (measured by the balanced accuracy and defect detection rate) compared to black-box AI ($\alpha = 0.05$).*

Hypothesis 3 (H3): *Explainable AI reduces variation in decision performance (measured by the variance in the balanced accuracy and defect detection rate) compared to black-box AI ($\alpha = 0.05$).*

Hypothesis 4 (H4): *Explainable AI increases the trust in model decisions (measured by the rate of correct model decisions that are not overruled by the user) compared to black-box AI ($\alpha = 0.05$).*

Table S18 summarizes the results from all three studies: (1) the manufacturing experiment at *Siemens*, (2) the medical experiment, and (3) the manufacturing experiments with non-experts from Amazon MTurk. We report the P-values for both the balanced accuracy and defect detection rate for hypotheses H1, H2, and H3. The statistical testing for hypotheses H1, H2, and H4 are based on one-sided Welch's t-tests. The statistical testing for Hypothesis H3 is based on Levene's test for equality of variances. As specified in our preregistration, we refrained from testing Hypothesis H1 in our manufacturing field experiment and our medical experiment. The reason is that we wanted sufficient power in our main treatment arms of interest (i.e., black-box AI versus explainable AI). All hypotheses except for Hypothesis H3 in the non-experts experiment and Hypotheses H2 and H3 for the disease detection rate in the medical experiment were confirmed at a significance level of $\alpha = 0.05$. The latter can be expected since missing a lung lesion has more serious consequences than erroneously believing a lung lesion is visible; thus, leading to conservative decision-making of radiologists. Therefore, we additionally inspected precision as a task performance metric. We find that radiologists augmented with explainable AI were significantly more precise in identifying lung lesions compared to radiologists with black-box AI (see Supplement G).

Table S18: Comparison of results against preregistered hypotheses.

	Study 1: Manufacturing	Study 2: Medical	Study 3: Non-experts
**H1** (BACC)	*not part of preregistration*	*not part of preregistration*	✓ ($P < 0.001$)
**H1** (DDR)	*not part of preregistration*	*not part of preregistration*	✓ ($P < 0.001$)
**H2** (BACC)	✓ ($P = 0.001$)	✓ ($P = 0.004$)	✓ ($P < 0.001$)
**H2** (DDR)	✓ ($P = 0.004$)	✗ ($P = 0.498$)	✓ ($P < 0.001$)
**H3** (BACC)	✓ ($P = 0.002$)	✓ ($P = 0.033$)	✗ ($P = 0.356$)
**H3** (DDR)	✓ ($P = 0.023$)	✗ ($P = 0.790$)	✗ ($P = 0.217$)
**H4**	✓ ($P = 0.011$)	✓ ($P = 0.001$)	✓ ($P = 0.009$)

Notes: The significance level was preregistered at $\alpha = 0.05$ and the marks denote whether the corresponding P-value was significant at this level. BACC refers to balanced accuracy and DDR to defect/disease detection rate.

Supplement L Post-experimental questionnaire

For post-hoc exploratory analyses, we asked participants to complete a questionnaire. The questions involved established constructs, such as self-reported task load [116], perceived usefulness [117], perceived ease of use [117], and self-reported trust [118]. We further asked participants about their previous experience and the perceived performance of the AI algorithm. In the manufacturing experiment, the questions were translated to German. In the medical experiment, the questions were adapted for the medical setting (for the exact wording, see our preregistration https://osf.io/69yqt). Participants in the non-experts experiment were asked to answer the questions from the viewpoint of a factory worker ("Imagine you work in a factory with a similar job task as you just did."). The results for all three studies are provided in Tables S19 to S23.

Table S19: Self-reported task load

Question	Study 1: Manufacturing		Study 2: Medical		Study 3: Non-experts	
	Human with black-box AI	Human with explainable AI	Human with black-box AI	Human with explainable AI	Human with black-box AI	Human with explainable AI
How mentally demanding was the task? (1 = very low, 7 = very high)	3.41 (1.37)	3.54 (1.17)	3.80 (1.34)	3.73 (1.60)	4.72 (1.58)	4.87 (1.62)
How physically demanding was the task? (1 = very low, 7 = very high)	3.09 (1.54)	2.85 (1.38)	2.18 (1.32)	2.52 (1.42)	4.09 (2.10)	3.95 (2.06)
How hurried or rushed was the pace of the task? (1 = very low, 7 = very high)	3.59 (1.10)	3.92 (1.23)	2.76 (1.35)	3.16 (1.66)	4.56 (1.55)	4.44 (1.68)
How successful were you in accomplishing what you were asked to do? (1 = very poor, 7 = very good)	5.27 (0.94)	5.81 (0.85)	5.64 (1.09)	5.57 (0.93)	5.68 (1.08)	5.87 (1.02)
How hard did you have to work to accomplish your level of performance? (1 = very low, 7 = very high)	4.00 (1.35)	4.00 (0.94)	3.33 (1.28)	3.36 (1.30)	5.18 (1.38)	5.29 (1.46)
How insecure, discouraged, irritated, stressed, and annoyed were you? (1 = very low, 7 = very high)	2.77 (1.31)	2.77 (1.58)	2.76 (1.28)	2.91 (1.43)	3.52 (1.98)	3.31 (1.95)

Notes: The table reports the average scores for the self-reported task load. Standard deviations are reported in parentheses.

Table S20: Perceived usefulness

Question	Study 1: Manufacturing		Study 2: Medical		Study 3: Non-experts	
	Human with black-box AI	Human with explainable AI	Human with black-box AI	Human with explainable AI	Human with black-box AI	Human with explainable AI
Using the Artificial Intelligence System in my job would enable me to accomplish tasks more quickly. (1 = very unlikely, 7 = extremely likely)	4.95 (1.70)	5.12 (1.37)	5.00 (1.40)	5.25 (1.06)	5.68 (1.02)	5.86 (1.08)
Using the Artificial Intelligence System would improve my job performance. (1 = very unlikely, 7 = extremely likely)	4.91 (1.54)	5.23 (0.99)	5.04 (1.24)	5.09 (1.14)	5.68 (1.04)	5.98 (1.06)
Using the Artificial Intelligence System in my job would increase my productivity. (1 = very unlikely, 7 = extremely likely)	4.95 (1.43)	4.96 (1.22)	5.04 (1.38)	5.39 (1.15)	5.71 (1.11)	5.98 (1.01)
Using the Artificial Intelligence System in my job would enhance my effectiveness on the job. (1 = very poor, 7 = very good)	5.05 (1.53)	4.88 (1.14)	4.98 (1.34)	5.20 (1.25)	5.66 (1.12)	5.91 (0.97)
Using the Artificial Intelligence System would make it easier to do my job. (1 = very unlikely, 7 = extremely likely)	5.18 (1.26)	5.08 (1.32)	4.93 (1.45)	5.23 (1.08)	5.65 (1.23)	6.01 (1.00)
I would find the Artificial Intelligence System useful in my job. (1 = very unlikely, 7 = extremely likely)	5.27 (1.42)	5.23 (1.24)	5.00 (1.41)	5.09 (1.22)	5.79 (1.09)	6.07 (1.04)

Notes: The table reports the average scores for the perceived usefulness of the AI algorithm. Standard deviations are reported in parentheses.

Table S21: Perceived ease of use

Question	Study 1: Manufacturing		Study 2: Medical		Study 3: Non-experts	
	Human with black-box AI	Human with explainable AI	Human with black-box AI	Human with explainable AI	Human with black-box AI	Human with explainable AI
Learning to operate the Artificial Intelligence System would be easy for me. (1 = very unlikely, 7 = extremely likely)	5.00 (1.45)	5.35 (1.20)	5.78 (0.88)	6.00 (0.86)	5.69 (1.11)	5.90 (0.95)
I would find it easy to get the Artificial Intelligence System to do what I want it to do. (1 = very unlikely, 7 = extremely likely)	4.05 (1.36)	4.46 (0.90)	4.87 (1.22)	5.09 (1.03)	5.61 (1.02)	5.78 (0.97)
My interaction with the Artificial Intelligence System would be clear and understandable. (1 = very unlikely, 7 = extremely likely)	5.09 (0.97)	5.27 (0.96)	5.16 (1.07)	5.20 (1.21)	5.65 (1.08)	5.99 (0.93)
I would find the Artificial Intelligence System to be flexible to interact with. (1 = very poor, 7 = very good)	4.82 (1.22)	5.00 (1.06)	4.82 (1.28)	4.84 (1.27)	5.36 (1.24)	5.55 (1.10)
It would be easy for me to become skillful at using the Artificial Intelligence System. (1 = very unlikely, 7 = extremely likely)	5.14 (1.21)	5.38 (0.85)	5.38 (1.21)	5.61 (0.84)	5.73 (0.97)	5.93 (0.99)
I would find the Artificial Intelligence System easy to use. (1 = very unlikely, 7 = extremely likely)	5.14 (0.94)	5.42 (1.03)	5.16 (1.17)	5.66 (0.83)	5.71 (1.03)	6.05 (0.96)

Notes: The table reports the average scores for the perceived ease of use of the AI algorithm. Standard deviations are reported in parentheses.

Table S22: Previous experience and perceived performance

Question	Study 1: Manufacturing		Study 2: Medical		Study 3: Non-experts	
	Human with black-box AI	Human with explainable AI	Human with black-box AI	Human with explainable AI	Human with black-box AI	Human with explainable AI
How strong would you consider your general IT skills? (1 = novice, 5 = expert)	2.86 (0.94)	3.08 (0.84)	3.47 (1.01)	3.77 (1.01)	3.48 (0.99)	3.48 (1.08)
How often do you interact with Artificial Intelligence in your job? (1 = very little, 5 = very much)	2.32 (1.13)	2.23 (1.11)	*not part of preregistration*		3.18 (1.31)	3.12 (1.32)
How familiar do you feel with Artificial Intelligence in general? (1 = very little, 5 = very much)	2.41 (1.10)	2.88 (1.07)	3.00 (1.07)	3.07 (0.95)	3.56 (0.98)	3.48 (0.93)
How well did the Artificial Intelligence System perform in comparison to your expectations? (1 = very poor, 7 = very good)	5.00 (1.57)	6.08 (1.06)	4.49 (1.41)	4.48 (1.47)	5.72 (1.01)	6.15 (0.85)
How likely is the Artificial Intelligence System to make a bad estimate? (1 = very unlikely, 7 = very likely)	3.55 (1.34)	3.04 (1.08)	3.93 (1.14)	4.02 (1.13)	3.25 (1.61)	2.93 (1.68)
Completing the quality inspections task has changed my opinion about Artificial Intelligence. (1 = strongly disagree, 7 = strongly agree)	4.64 (1.36)	4.08 (1.38)	*not part of preregistration*		4.68 (1.65)	4.83 (1.61)
How much did you rely on the Artificial Intelligence System? (1 = very little, 7 = very much)	4.32 (1.46)	4.77 (1.37)	*not part of preregistration*		4.96 (1.43)	5.54 (1.38)
The Artificial Intelligence System provides clear explanations for its outputs. (1 = strongly disagree, 7 = strongly agree)	4.59 (1.33)	5.04 (1.00)	*not part of preregistration*		4.99 (1.54)	5.71 (1.20)

Notes: The table reports the average scores for previous experience and the perceived performance of the AI algorithm. Standard deviations are reported in parentheses.

Table S23: Self-reported trust in AI algorithm

Question	Study 1: Manufacturing		Study 2: Medical		Study 3: Non-experts	
	Human with black-box AI	Human with explainable AI	Human with black-box AI	Human with explainable AI	Human with black-box AI	Human with explainable AI
The Artificial Intelligence System is deceptive. (1 = strongly disagree, 7 = strongly agree)	3.27 (1.20)	2.88 (1.18)	3.31 (1.41)	3.39 (1.28)	3.79 (2.04)	3.72 (2.16)
I am suspicious of the Artificial Intelligence System's intent, action, or outputs. (1 = strongly disagree, 7 = strongly agree)	2.91 (1.27)	2.62 (0.98)	3.09 (1.69)	2.98 (1.56)	3.88 (2.04)	3.69 (2.15)
The Artificial Intelligence System's actions will have a harmful outcome. (1 = strongly disagree, 7 = strongly agree)	3.09 (1.41)	2.65 (1.09)	3.38 (1.51)	3.34 (1.43)	3.73 (2.04)	3.48 (2.08)
I am confident in the Artificial Intelligence System. (1 = very poor, 7 = very good)	4.91 (1.06)	4.96 (1.15)	4.04 (1.31)	4.34 (1.22)	5.52 (1.20)	5.89 (0.97)
The Artificial Intelligence System is reliable. (1 = strongly disagree, 7 = strongly agree)	4.95 (0.84)	5.27 (1.04)	4.07 (1.39)	4.27 (1.09)	5.63 (1.10)	5.93 (0.85)
I can trust the Artificial Intelligence System. (1 = strongly disagree, 7 = strongly agree)	4.86 (0.77)	5.04 (0.92)	3.87 (1.39)	4.07 (1.11)	5.66 (1.06)	5.80 (0.94)

Notes: The table reports the average scores for the self-reported trust in the AI algorithm. Standard deviations are reported in parentheses.

References

[1] Guidotti, R., Monreale, A., Ruggieri, S., Turini, F., Giannotti, F. & Pedreschi, D. A survey of methods for explaining black box models. *ACM Computing Surveys* **51**, 1–42 (2018).

[2] Samek, W., Montavon, G., Vedaldi, A., Hansen, L. K. & Müller, K.-R. *Explainable AI: Interpreting, Explaining and Visualizing Deep Learning* (Springer Nature, 2019).

[3] Linardatos, P., Papastefanopoulos, V. & Kotsiantis, S. Explainable AI: A review of machine learning interpretability methods. *Entropy* **23** (2020).

[4] Samek, W., Montavon, G., Lapuschkin, S., Anders, C. J. & Muller, K.-R. Explaining deep neural networks and beyond: A review of methods and applications. *Proceedings of the IEEE* **109**, 247–278 (2021).

[5] Rudin, C. Stop explaining black box machine learning models for high stakes decisions and use interpretable models instead. *Nature Machine Intelligence* **1**, 206–215 (2019).

[6] Nelder, J. A. & Wedderburn, R. W. M. Generalized linear models. *Journal of the Royal Statistical Society. Series A (General)* **135**, 370–384 (1972).

[7] Hastie, T. & Tibshirani, R. *Generalized additive models* (Chapman & Hall/CRC, 1990).

[8] Lou, Y., Caruana, R. & Gehrke, J. Intelligible models for classification and regression. In *ACM SIGKDD International Conference on Knowledge Discovery and Data Mining* (2012).

[9] Kraus, M., Tschernutter, D., Weinzierl, S. & Zschech, P. Interpretable generalized additive neural networks. *European Journal of Operational Research* **317**, 303–316 (2024).

[10] Molnar, C. *Interpretable machine learning: A guide for making Black Box Models interpretable* (Lulu. com, 2019).

[11] Ribeiro, M. T., Singh, S. & Guestrin, C. Why should I trust you? Explaining the predictions of any classifier. In *ACM SIGKDD International Conference on Knowledge Discovery and Data Mining* (2016).

[12] Lundberg, S. & Lee, S.-I. A unified approach to interpreting model predictions. In *Advances in Neural Information Processing Systems* (2017).

[13] Shapley, L. S. A value for n-person games. In *Contributions to the Theory of Games*, Annals of Mathematics Studies, 307–318 (Princeton University Press, 1953).

[14] Selvaraju, R. R., Cogswell, M., Das, A., Vedantam, R., Parikh, D. & Batra, D. Grad-CAM: Visual explanations from deep networks via gradient-based localization. In *IEEE International Conference on Computer Vision*, 618–626 (2017).

[15] Hansen, K., Baehrens, D., Schroeter, T., Rupp, M. & Müller, K.-R. Visual interpretation of kernel-based prediction models. *Molecular Informatics* **30**, 817–826 (2011).

[16] Buhrmester, V., Münch, D. & Arens, M. Analysis of explainers of black box deep neural networks for computer vision: A survey. *Machine Learning and Knowledge Extraction* **3**, 966–989 (2021).

[17] Ibrahim, R. & Shafiq, M. O. Explainable convolutional neural networks: A taxonomy, review, and future directions. *ACM Computing Surveys* **55**, 1–37 (2023).

[18] Bastani, O., Kim, C. & Bastani, H. Interpreting blackbox models via model extraction. Preprint at *arXiv* https://arxiv.org/abs/1705.08504 (2017).

[19] Žlahtič, B., Završnik, J., Blažun Vošner, H. & Kokol, P. Transferring black-box decision making to a white-box model. *Electronics* **13**, 1895 (2024).

[20] Žlahtič, B., Završnik, J., Blažun Vošner, H., Kokol, P., Šuran, D. & Završnik, T. Agile machine learning model development using data canyons in medicine: A step towards explainable artificial intelligence and flexible expert-based model improvement. *Applied Sciences* **13**, 8329 (2023).

[21] Zeiler, M. D. & Fergus, R. Visualizing and understanding convolutional networks. In *European Conference on Computer Vision*, 8689 (2014).

[22] Simonyan, K., Vedaldi, A. & Zisserman, A. Deep inside convolutional networks: Visualising image classification models and saliency maps. In *Workshop at International Conference on Learning Representations* (2014).

[23] Bach, S., Binder, A., Montavon, G., Klauschen, F., Müller, K.-R. & Samek, W. On pixel-wise explanations for non-linear classifier decisions by layer-wise relevance propagation. *PLOS ONE* **10**, e0130140 (2015).

[24] Sundararajan, M., Taly, A. & Yan, Q. Axiomatic attribution for deep networks. In *International Conference on Machine Learning*, 70 (2017).

[25] Shrikumar, A., Greenside, P. & Kundaje, A. Learning important features through propagating activation differences. In *International Conference on Machine Learning*, 70 (2017).

[26] Zhou, B., Khosla, A., Lapedriza, A., Oliva, A. & Torralba, A. Learning deep features for discriminative localization. In *IEEE Conference on Computer Vision and Pattern Recognition* (2016).

[27] Chattopadhay, A., Sarkar, A., Howlader, P. & Balasubramanian, V. N. Grad-CAM++: Generalized gradient-based visual explanations for deep convolutional networks. In *IEEE Winter Conference on Applications of Computer Vision*, 839–847 (2018).

[28] Bany Muhammad, M. & Yeasin, M. Eigen-CAM: Visual explanations for deep convolutional neural networks. *SN Computer Science* **2** (2021).

[29] Verma, S., Dickerson, J. P. & Hines, K. E. Counterfactual explanations for machine learning: A review. Preprint at *arXiv* https://doi.org/10.48550/arXiv.2010.10596 (2020).

[30] Bergmann, P., Löwe, S., Fauser, M., Sattlegger, D. & Steger, C. Improving unsupervised defect segmentation by applying structural similarity to autoencoders. Preprint at *arXiv* https://doi.org/10.48550/arXiv.1807.02011 (2019).

[31] Zipfel, J., Verworner, F., Fischer, M., Wieland, U., Kraus, M. & Zschech, P. Anomaly detection for industrial quality assurance: A comparative evaluation of unsupervised deep learning models. *Computers & Industrial Engineering* **177**, 109045 (2023).

[32] Liu, J., Xie, G., Wang, J., Li, S., Wang, C., Zheng, F. & Jin, Y. Deep industrial image anomaly detection: A survey. *Machine Intelligence Research* **21**, 104–135 (2024).

[33] Doshi-Velez, F. & Kim, B. Towards a rigorous science of interpretable machine learning. Preprint at *arXiv* https://doi.org/10.48550/arXiv.1702.08608 (2017).

[34] Plumb, G., Molitor, D. & Talwalkar, A. S. Model agnostic supervised local explanations. In *Advances in Neural Information Processing Systems* (2018).

[35] Hooker, S., Erhan, D., Kindermans, P.-J. & Kim, B. A benchmark for interpretability methods in deep neural networks. In *Advances in Neural Information Processing Systems* (2019).

[36] Saporta, A., Gui, X., Agrawal, A., Pareek, A., Truong, S. Q. H., Nguyen, C. D. T., Ngo, V.-D., Seekins, J., Blankenberg, F. G., Ng, A. Y., Lungren, M. P. & Rajpurkar, P. Benchmarking saliency methods for chest X-ray interpretation. *Nature Machine Intelligence* **4**, 867–878 (2022).

[37] Alvarez-Melis, D. & Jaakkola, T. S. On the robustness of interpretability methods. Preprint at *arXiv* https://doi.org/10.48550/arXiv.1806.08049 (2018).

[38] Narayanan, M., Chen, E., He, J., Kim, B., Gershman, S. & Doshi-Velez, F. How do humans understand explanations from machine learning systems? An evaluation of the human-interpretability of explanation. Preprint at *arXiv* https://doi.org/10.48550/arXiv.1802.00682 (2018).

[39] Jesus, S., Belém, C., Balayan, V., Bento, J., Saleiro, P., Bizarro, P. & Gama, J. How can I choose an explainer? In *ACM Conference on Fairness, Accountability, and Transparency* (2021).

[40] Amarasinghe, K., Rodolfa, K. T., Jesus, S., Chen, V., Balayan, V., Saleiro, P., Bizarro, P., Talwalkar, A. & Ghani, R. On the importance of application-grounded experimental design for evaluating explainable ML methods. *AAAI Conference on Artificial Intelligence* **38** (2024).

[41] Liu, M., Shi, J., Li, Z., Li, C., Zhu, J. & Liu, S. Towards better analysis of deep convolutional neural networks. *IEEE Transactions on Visualization and Computer Graphics* **23**, 91–100 (2017).

[42] Ming, Y., Cao, S., Zhang, R., Li, Z., Chen, Y., Song, Y. & Qu, H. Understanding hidden memories of recurrent neural networks. In *IEEE Conference on Visual Analytics Science and Technology* (2017).

[43] Pezzotti, N., Hollt, T., van Gemert, J., Lelieveldt, B. P. F., Eisemann, E. & Vilanova, A. DeepEyes: Progressive visual analytics for designing deep neural networks. *IEEE Transactions on Visualization and Computer Graphics* **24**, 98–108 (2018).

[44] Strobelt, H., Gehrmann, S., Pfister, H. & Rush, A. M. LSTMVis: A tool for visual analysis of hidden state dynamics in recurrent neural networks. *IEEE Transactions on Visualization and Computer Graphics* **24**, 667–676 (2018).

[45] Fügener, A., Grahl, J., Gupta, A. & Ketter, W. Will humans-in-the-loop become borgs? Merits and pitfalls of working with AI. *MIS Quarterly* **45**, 1527–1556 (2021).

[46] Fügener, A., Grahl, J., Gupta, A. & Ketter, W. Cognitive challenges in human–artificial intelligence collaboration: Investigating the path toward productive delegation. *Information Systems Research* **33**, 678–696 (2022).

[47] Fügener, A., Gupta, A., Grahl, J., Ketter, W. & Taudien, A. Exploring user heterogeneity in human delegation behavior towards AI. In *International Conference on Information Systems* (2021).

[48] Candrian, C. & Scherer, A. Rise of the machines: Delegating decisions to autonomous AI. *Computers in Human Behavior* **134**, 107308 (2022).

[49] Bauer, K., von Zahn, M. & Hinz, O. Please take over: XAI, delegation of authority, and domain knowledge. Preprint at *SSRN* `https://doi.org/10.2139/ssrn.4512594` (2023).

[50] Dietvorst, B. J., Simmons, J. P. & Massey, C. Algorithm aversion: People erroneously avoid algorithms after seeing them err. *Journal of Experimental Psychology: General* **144**, 114–126 (2015).

[51] Dietvorst, B. J., Simmons, J. P. & Massey, C. Overcoming algorithm aversion: People will use imperfect algorithms if they can (even slightly) modify them. *Management Science* **64**, 1155–1170 (2018).

[52] Dietvorst, B. J. & Bharti, S. People reject algorithms in uncertain decision domains because they have diminishing sensitivity to forecasting error. *Psychological Science* **31**, 1302–1314 (2020).

[53] Burton, J. W., Stein, M.-K. & Jensen, T. B. A systematic review of algorithm aversion in augmented decision making. *Journal of Behavioral Decision Making* **33**, 220–239 (2020).

[54] Jessica Ochmann, Leonard Michels, Sandra Zilker, Verena Tiefenbeck & Sven Laumer. The influence of algorithm aversion and anthropomorphic agent design on the acceptance of AI-based job recommendations. In *International Conference on Information Systems* (2020).

[55] Hou, Y. T.-Y. & Jung, M. F. Who is the expert? Reconciling algorithm aversion and algorithm appreciation in AI-supported decision making. *ACM on Human-Computer Interaction* **5**, 477 (2021).

[56] Bogert, E., Schecter, A. & Watson, R. T. Humans rely more on algorithms than social influence as a task becomes more difficult. *Scientific Reports* **11**, 8028 (2021).

[57] Cadario, R., Longoni, C. & Morewedge, C. K. Understanding, explaining, and utilizing medical artificial intelligence. *Nature Human Behaviour* **5**, 1636–1642 (2021).

[58] Sun, J., Zhang, D. J., Hu, H. & van Mieghem, J. A. Predicting human discretion to adjust algorithmic prescription: A large-scale field experiment in warehouse operations. *Management Science* **68**, 846–865 (2022).

[59] Kawaguchi, K. When will workers follow an algorithm? A field experiment with a retail business. *Management Science* **67**, 1670–1695 (2021).

[60] Castelo, N., Bos, M. W. & Lehmann, D. R. Task-dependent algorithm aversion. *Journal of Marketing Research* **56**, 809–825 (2019).

[61] Yeomans, M., Shah, A., Mullainathan, S. & Kleinberg, J. Making sense of recommendations. *Journal of Behavioral Decision Making* **32**, 403–414 (2019).

[62] Jiang, J., Kahai, S. & Yang, M. Who needs explanation and when? Juggling explainable AI and user epistemic uncertainty. *International Journal of Human-Computer Studies* **165**, 102839 (2022).

[63] Ben David, D., Resheff, Y. S. & Tron, T. Explainable AI and adoption of financial algorithmic advisors. In *AAAI/ACM Conference on AI, Ethics, and Society*, 390–400 (2021).

[64] Fleiß, J., Bäck, E. & Thalmann, S. Mitigating algorithm aversion in recruiting: A study on explainable AI for conversational agents. *ACM SIGMIS Database: the DATABASE for Advances in Information Systems* **55**, 56–87 (2024).

[65] Choung, H., David, P. & Ross, A. Trust in AI and its role in the acceptance of AI technologies. *International Journal of Human–Computer Interaction* **39**, 1727–1739 (2023).

[66] Cai, C. J., Reif, E., Hegde, N., Hipp, J., Kim, B., Smilkov, D., Wattenberg, M., Viegas, F., Corrado, G. S., Stumpe, M. C. & Terry, M. Human-centered tools for coping with imperfect algorithms during medical decision-making. In *CHI Conference on Human Factors in Computing Systems* (2019).

[67] Nourani, M., Kabir, S., Mohseni, S. & Ragan, E. D. The effects of meaningful and meaningless explanations on trust and perceived system accuracy in intelligent systems. *AAAI Conference on Human Computation and Crowdsourcing* **7** (2019).

[68] Nourani, M., King, J. & Ragan, E. The role of domain expertise in user trust and the impact of first impressions with intelligent systems. *AAAI Conference on Human Computation and Crowdsourcing* **8**, 112–121 (2020).

[69] Sabol, P., Sinčák, P., Hartono, P., Kočan, P., Benetinová, Z., Blichárová, A., Verbóová, Ľ., Štammová, E., Sabolová-Fabianová, A. & Jašková, A. Explainable classifier for improving the accountability in decision-making for colorectal cancer diagnosis from histopathological images. *Journal of Biomedical Informatics* **109**, 103523 (2020).

[70] Branley-Bell, D., Whitworth, R. & Coventry, L. User trust and understanding of explainable AI: Exploring algorithm visualisations and user biases. In *Human-Computer Interaction. Human Values and Quality of Life*, 12183, 382–399 (2020).

[71] Zhang, Y., Liao, Q. V. & Bellamy, R. K. E. Effect of confidence and explanation on accuracy and trust calibration in AI-assisted decision making. In *Conference on Fairness, Accountability, and Transparency*, 295–305 (2020).

[72] Folke, T., Yang, S. C.-H., Anderson, S. & Shafto, P. Explainable AI for medical imaging: explaining pneumothorax diagnoses with Bayesian teaching. In *Artificial Intelligence and Machine Learning for Multi-Domain Operations Applications III*, 83 (2021).

[73] Lancaster Farrell, C.-J. Explainability does not improve biochemistry staff trust in artificial intelligence-based decision support. *Annals of Clinical Biochemistry* **59**, 447–449 (2022).

[74] Panigutti, C., Beretta, A., Giannotti, F. & Pedreschi, D. Understanding the impact of explanations on advice-taking: a user study for AI-based clinical decision support systems. In *CHI Conference on Human Factors in Computing Systems* (2022).

[75] Panigutti, C., Beretta, A., Fadda, D., Giannotti, F., Pedreschi, D., Perotti, A. & Rinzivillo, S. Co-design of human-centered, explainable AI for clinical decision support. *ACM Transactions on Interactive Intelligent Systems* **13** (2023).

[76] Leichtmann, B., Humer, C., Hinterreiter, A., Streit, M. & Mara, M. Effects of explainable artificial intelligence on trust and human behavior in a high-risk decision task. *Computers in Human Behavior* **139**, 107539 (2023).

[77] Sivaraman, V., Bukowski, L. A., Levin, J., Kahn, J. M. & Perer, A. Ignore, trust, or negotiate: Understanding clinician acceptance of AI-based treatment recommendations in health care. In *CHI Conference on Human Factors in Computing Systems* (2023).

[78] Buçinca, Z., Malaya, M. B. & Gajos, K. Z. To trust or to think: Cognitive forcing functions can reduce overreliance on AI in AI-assisted decision-making. *ACM on Human-Computer Interaction* **5**, 188 (2021).

[79] Vasconcelos, H., Jörke, M., Grunde-McLaughlin, M., Gerstenberg, T., Bernstein, M. S. & Krishna, R. Explanations can reduce overreliance on AI systems during decision-making. *ACM on Human-Computer Interaction* **7**, 129 (2023).

[80] Bansal, G., Wu, T., Zhou, J., Fok, R., Nushi, B., Kamar, E., Ribeiro, M. T. & Weld, D. Does the whole exceed its parts? The effect of AI explanations on complementary team performance. In *CHI Conference on Human Factors in Computing Systems* (2021).

[81] Chen, V., Liao, Q. V., Wortman Vaughan, J. & Bansal, G. Understanding the role of human intuition on reliance in human-AI decision-making with explanations. *ACM on Human-Computer Interaction* **7**, 370 (2023).

[82] Lee, J. D. & See, K. A. Trust in automation: designing for appropriate reliance. *Human factors* **46**, 50–80 (2004).

[83] Green, B. & Chen, Y. The principles and limits of algorithm-in-the-loop decision making. *ACM on Human-Computer Interaction* **3**, 50 (2019).

[84] Lage, I., Chen, E., He, J., Narayanan, M., Kim, B., Gershman, S. J. & Doshi-Velez, F. Human evaluation of models built for interpretability. *AAAI Conference on Human Computation and Crowdsourcing* **7**, 59–67 (2019).

[85] Lai, V. & Tan, C. On human predictions with explanations and predictions of machine learning models. In *Conference on Fairness, Accountability, and Transparency* (2019).

[86] Cai, C. J., Jongejan, J. & Holbrook, J. The effects of example-based explanations in a machine learning interface. In *International Conference on Intelligent User Interfaces* (2019).

[87] Buçinca, Z., Lin, P., Gajos, K. Z. & Glassman, E. L. Proxy tasks and subjective measures can be misleading in evaluating explainable AI systems. In *International Conference on Intelligent User Interfaces* (2020).

[88] Carton, S., Mei, Q. & Resnick, P. Feature-based explanations don't help people detect misclassifications of online toxicity. *International AAAI Conference on Web and Social Media* **14** (2020).

[89] Lai, V., Liu, H. & Tan, C. "Why is 'Chicago' deceptive?" Towards building model-driven tutorials for humans. In *CHI Conference on Human Factors in Computing Systems* (2020).

[90] Yang, F., Huang, Z., Scholtz, J. & Arendt, D. L. How do visual explanations foster end users' appropriate trust in machine learning? In *International Conference on Intelligent User Interfaces* (2020).

[91] Chu, E., Roy, D. & Andreas, J. Are visual explanations useful? A case study in model-in-the-loop prediction. Preprint at *arXiv* https://doi.org/10.48550/arXiv.2007.12248 (2020).

[92] Alqaraawi, A., Schuessler, M., Weiß, P., Costanza, E. & Berthouze, N. Evaluating saliency map explanations for convolutional neural networks. In *International Conference on Intelligent User Interfaces* (2020).

[93] Wang, X. & Yin, M. Are explanations helpful? A comparative study of the effects of explanations in AI-assisted decision-making. In *International Conference on Intelligent User Interfaces* (2021).

[94] Poursabzi-Sangdeh, F., Goldstein, D. G., Hofman, J. M., Wortman Vaughan, J. W. & Wallach, H. Manipulating and measuring model interpretability. In *CHI Conference on Human Factors in Computing Systems* (2021).

[95] van der Waa, J., Nieuwburg, E., Cremers, A. & Neerincx, M. Evaluating XAI: A comparison of rule-based and example-based explanations. *Artificial Intelligence* **291**, 103404 (2021).

[96] Alufaisan, Y., Marusich, L. R., Bakdash, J. Z., Zhou, Y. & Kantarcioglu, M. Does explainable artificial intelligence improve human decision-making? *AAAI Conference on Artificial Intelligence* **35** (2021).

[97] Nourani, M., Roy, C., Block, J. E., Honeycutt, D. R., Rahman, T., Ragan, E. & Gogate, V. Anchoring bias affects mental model formation and user reliance in explainable AI systems. In *International Conference on Intelligent User Interfaces*, 340–350 (2021).

[98] Kim, S. S. Y., Meister, N., Ramaswamy, V. V., Fong, R. & Russakovsky, O. Hive: Evaluating the human interpretability of visual explanations. In *European Conference on Computer Vision*, 13672 (2022).

[99] Leichtmann, B., Hinterreiter, A., Humer, C., Streit, M. & Mara, M. Explainable artificial intelligence improves human decision-making: Results from a mushroom picking experiment at a public art festival. *International Journal of Human–Computer Interaction* 1–18 (2023).

[100] Müller, R., Reindel, D. F. & Stadtfeld, Y. D. The benefits and costs of explainable artificial intelligence in visual quality control: Evidence from fault detection performance and eye movements. *Human Factors and Ergonomics in Manufacturing & Service Industries* (2024).

[101] Spitzer, P., Holstein, J., Morrison, K., Holstein, K., Satzger, G. & Kühl, N. Don't be fooled: The misinformation effect of explanations in human-AI collaboration. Preprint at *arXiv* https://arxiv.org/abs/2409.12809 (2024).

[102] Lundberg, S. M., Erion, G., Chen, H., DeGrave, A., Prutkin, J. M., Nair, B., Katz, R., Himmelfarb, J., Bansal, N. & Lee, S.-I. From local explanations to global understanding with explainable AI for trees. *Nature Machine Intelligence* **2**, 56–67 (2020).

[103] Das, N. *et al.* Collaboration between explainable artificial intelligence and pulmonologists improves the accuracy of pulmonary function test interpretation. *European Respiratory Journal* **61**, 2201720 (2023).

[104] Gaube, S., Suresh, H., Raue, M., Lermer, E., Koch, T. K., Hudecek, M. F. C., Ackery, A. D., Grover, S. C., Coughlin, J. F., Frey, D., Kitamura, F. C., Ghassemi, M. & Colak, E. Non-task expert physicians benefit from correct explainable AI advice when reviewing X-rays. *Scientific Reports* **13**, 1383 (2023).

[105] Jabbour, S., Fouhey, D., Shepard, S., Valley, T. S., Kazerooni, E. A., Banovic, N., Wiens, J. & Sjoding, M. W. Measuring the impact of AI in the diagnosis of hospitalized patients: A randomized clinical vignette survey study. *JAMA* **330**, 2275–2284 (2023).

[106] Metta, C., Beretta, A., Guidotti, R., Yin, Y., Gallinari, P., Rinzivillo, S. & Giannotti, F. Improving trust and confidence in medical skin lesion diagnosis through explainable deep learning. *International Journal of Data Science and Analytics* (2023).

[107] Nagendran, M., Festor, P., Komorowski, M., Gordon, A. C. & Faisal, A. A. Quantifying the impact of AI recommendations with explanations on prescription decision making. *npj Digital Medicine* **6**, 206 (2023).

[108] Irvin, J. *et al.* CheXpert: A large chest radiograph dataset with uncertainty labels and expert comparison. *AAAI Conference on Artificial Intelligence* **33** (2019).

[109] UTSouthwestern Medical Center. Pulmonary nodules and lung lesions (Accessed 04/09/24). URL https://utswmed.org/conditions-treatments/pulmonary-nodules-and-lung-lesions/.

[110] Oestmann, J. W., Greene, R., Kushner, D. C., Bourgouin, P. M., Linetsky, L. & Llewellyn, H. J. Lung lesions: correlation between viewing time and detection. *Radiology* **166**, 451–453 (1988).

[111] Pimentel, M. A., Clifton, D. A., Clifton, L. & Tarassenko, L. A review of novelty detection. *Signal Processing* **99**, 215–249 (2014).

[112] Bergmann, P., Fauser, M., Sattlegger, D. & Steger, C. MVTec AD — A comprehensive real-world dataset for unsupervised anomaly detection. In *IEEE/CVF Conference on Computer Vision and Pattern Recognition* (2019).

[113] Wang, Z., Bovik, A. C., Sheikh, H. R. & Simoncelli, E. P. Image quality assessment: From error visibility to structural similarity. *IEEE Transactions on Image Processing* **13**, 600–612 (2004).

[114] Huang, G., Liu, Z., van der Maaten, L. & Weinberger, K. Q. Densely connected convolutional networks. In *IEEE Conference on Computer Vision and Pattern Recognition*, 4700–4708 (2017).

[115] Peer, E., Vosgerau, J. & Acquisti, A. Reputation as a sufficient condition for data quality on Amazon Mechanical Turk. *Behavior Research Methods* **46**, 1023–1031 (2013).

[116] NASA. Nasa Task Load Index (TLX) (1986). URL https://humansystems.arc.nasa.gov/groups/TLX/downloads/TLX.pdf.

[117] Davis, F. D. Perceived usefulness, perceived ease of use, and user acceptance of information technology. *MIS Quarterly* **13**, 319–340 (1989).

[118] Jian, J.-Y., Bisantz, A. M. & Drury, C. G. Foundations for an empirically determined scale of trust in automated systems. *International Journal of Cognitive Ergonomics* **4**, 53–71 (2000).
